# Prognostic value and experimental validation of atherosclerosis-derived pathogenic genes in colorectal cancer

**DOI:** 10.3389/fonc.2025.1728087

**Published:** 2026-01-12

**Authors:** Yuqing Li, Jinhong Wei, Yuanyuan Xu, Zhenyu Wu, Saiqi He, Yuhang Zhu, Wen Ni, Di Zhang, Huiya Xu, Chuanjie Zhang, Aijun Zhou, Tong Shen, Jianming Li

**Affiliations:** 1 Department of Pathology, Sun Yat-Sen Memorial Hospital, Sun Yat-Sen University, Guangzhou, China; 2 Department of Pathology, Soochow University Medical School,, Suzhou, China; 3 Department of Anesthesiology, Affiliated Hospital of Zunyi Medical University, Zunyi, China; 4 Key Laboratory of Brain Function and Brain Disease Prevention and Treatment of Guizhou Province, Affiliated Hospital of Zunyi Medical University, Zunyi, China

**Keywords:** colorectal cancer, atherosclerosis, bioinformatics, risk model, tumor immune microenvironment

## Abstract

**Objectives:**

Colorectal cancer (CRC) and atherosclerosis (AS) share pathological phenotypes and clinical links, but their shared pathogenic mechanisms are unclear. This study aimed to identify shared genetic drivers, construct a CRC risk model using AS-related genes, and validate expression via multi-omics.

**Methods:**

Transcriptomic data from The Cancer Genome Atlas and Gene Expression Omnibus were analyzed. Core gene modules associated with CRC and AS were screened using weighted gene co-expression network analysis and differentially expressed genes with significant expression differences between CRC tissues and normal tissues were identified through differential analysis. The intersection of these three sets of genes was taken to determine the overlapping genes. A prognostic model with 6 key genes (*CDC25C*, *HMMR*, *KPNA2*, *PRR11*, *PALB2*, and *TKT*) was built via univariate Cox and least absolute shrinkage and selection operator analyses. High/low-risk groups underwent Gene Set Enrichment Analysis (GSEA), immune infiltration, and immune checkpoint analyses. Multi-omics characterized gene expression/localization, validated by reverse transcription-quantitative polymerase chain reaction, Western blotting, and immunohistochemistry.

**Results:**

The model showed reliable predictive performance. Low-risk groups had enriched activated dendritic cells and follicular helper T cells; high-risk groups featured memory B cells and resting mast cells. Most genes overexpressed in lesions, except *PRR11* (higher in normal tissues). Experiments confirmed *HMMR*/*PALB2* overexpression in CRC and three AS genes elevated in AS lesions.

**Conclusion:**

A CRC risk model based on 6 AS-related genes was developed, identifying 3 novel AS genes. It highlights shared genetic factors, offering prognostic biomarkers for both diseases and insights into their interconnected mechanisms.

## 1 Introduction

Colorectal Cancer (CRC) is one of the leading causes of cancer-related mortality worldwide, ranking second in global cancer deaths. Its typical phenotypes include metabolic reprogramming, immune dysregulation, and genetic mutations (1). In recent years, a growing body of research has demonstrated that chronic diseases or conditions such as diabetes and metabolic syndrome share common pathogenic mechanisms with CRC (2–4). Notably, atherosclerosis (AS), a cardiovascular disease characterized by endothelial injury and cholesterol deposition, exhibits significant phenotypic overlaps with CRC in terms of lipid metabolism reprogramming and remodeling of the immune microenvironment (5).

At the lipid metabolic level, fatty acid synthase (*FASN*)-mediated dysregulated fatty acid metabolism in CRC promotes tumor proliferation, metastasis, and immune microenvironment disorder. Meanwhile, aberrant cholesterol metabolism drives carcinogenesis via multiple pathways (6, 7). Similarly, in AS, disrupted fatty acid metabolism leads to saturated fatty acid accumulation within vulnerable plaques, exacerbating vascular inflammation, and dysfunctional cholesterol metabolism directly accelerates plaque progression (8, 9).

During immune microenvironment remodeling, tumor-associated neutrophils in CRC secrete IL-8 and TNF-α to establish a pro-tumorigenic niche that facilitates immune evasion (10). Moreover, increased regulatory T cells (Tregs) and Th1/Th2 imbalance in CRC correlate with immunosuppression and tumor progression (11). Similarly, neutrophils in AS sustain chronic inflammation via the release of inflammatory mediators, while T lymphocytes recruited by chemokine cascades in AS induce analogous immune dysregulation, accelerating plaque development (12).

Clinical evidence further supports this association: A 2020 study of 29,610 newly diagnosed CRC patients revealed a 9.2% AS prevalence, particularly elevated in male and urban subgroups (13). Another investigation reported coronary artery calcification (CAC)—indicating subclinical AS—in 36.6% of 300 CRC patients, with CAC comorbidity significantly increasing all-cause mortality risk (14). On the other hand, the Multi-Ethnic Study of Atherosclerotic Disease (MESA) showed that patients with severe coronary artery calcification (CAC > 400) had a 2.2-fold increased risk of CRC (subdistribution hazard ratio [SHR] = 2.1, 95% confidence interval [CI]: 1.1–4.7) (15). A longitudinal study in Korea also found that the presence of carotid plaques was a predictor of advanced colorectal neoplasia (odds ratio [OR] = 2.1, 95% CI: 1.4–3.2) (16). These synergistic comorbidities demand mechanistic explanations at the molecular level.

This study aims to clarify the genetic basis of CRC-AS comorbidity, with findings expected to translate into clinical value at multiple levels. On the one hand, we innovatively take AS (a common non-tumor disease sharing multiple pathogenic mechanisms with CRC) as the entry point. By adopting an integrated multi-omics strategy (genomic, transcriptomic, and other data), we break the paradigm of previous CRC-focused studies. Leveraging multi-omics’ systematic analysis, we accurately identified shared pathogenic genes/pathways between the two diseases and constructed a CRC risk model. This provides a novel perspective for understanding their intrinsic mechanisms and offers specific, reliable biomarkers for CRC prognostic assessment and risk stratification. On the other hand, these multi-omics-identified shared genes/pathways unravel the molecular logic of comorbidity. They also clarify potential targets for dual-disease intervention, laying a solid foundation for developing the “one-target, dual-treatment” strategy. Ultimately, this study connects the “disease association - gene screening - clinical translation” chain via multi-omics. It is expected to innovate CRC-AS comorbidity management, shifting from passive complication handling to active risk prediction and synergistic intervention.

## 2 Materials and methods

### 2.1 Study design and data sources

**Figure 1:**
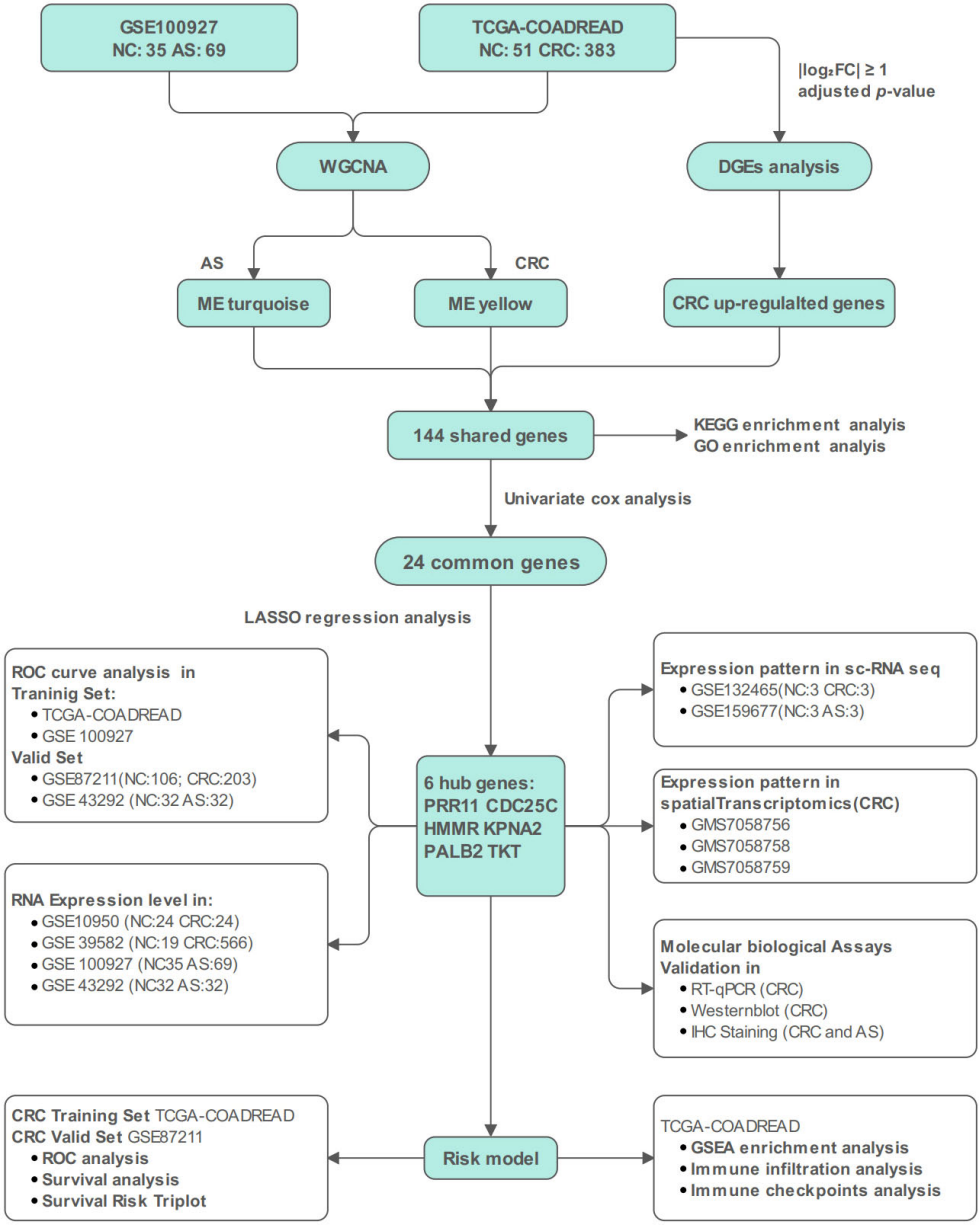
The flow chart of this study. NC, Normal Control; AS, Atherosclerosis; CRC, Colorectal Cancer; TCGA-COADREAD, The Cancer Genome Atlas-Colon Adenocarcinoma and Rectosigmoid Junction Adenocarcinoma; WGCNA, Weighted Gene Co-expression Network Analysis; DGEs, Differentially Expressed Genes; KEGG, Kyoto Encyclopedia of Genes and Genomes; GO, Gene Ontology; LASSO, Least Absolute Shrinkage and Selection Operator; ROC, Receiver Operating Characteristic; sc-RNA seq, Single-Cell RNA Sequencing; RT-qPCR, Reverse Transcription-Quantitative Polymerase Chain Reaction; IHC, Immunohistochemistry; GSEA, Gene Set Enrichment Analysis.

The technical workflow of this study is illustrated in **Figure 1**. Detailed information on the relevant datasets is summarized in **Table 1** and **Supplementary Table S1**.

**Table 1:** Dataset IDs, analytical applications and sample sizes designed in this study.

Dataset ID	Analytical applications	Control count	Case count
TCGA-COADREAD	WGCNA analysis; DGEs analysis; machine learning (univariate Cox and LASSO analysis); training set for risk model construction; immune infiltration analysis and immune checkpoint analysis of the risk model; single-gene ROC analysis	51	383
GSE87211	Validation set for CRC risk model; validation set for single-gene ROC analysis	160	203
GSE10950, GSE39582	Validation set for single-gene expression levels in normal tissues vs. CRC tissues	24	24
GSE132465	Single-cell transcriptomic analysis of CRC	3	3
GSE225857	Spatial transcriptomic analysis of CRC	0	3
GSE100927	WGCNA analysis; single-gene ROC analysis; validation set for single-gene expression levels in normal tissues vs. AS tissues	35	69
GSE43292	Single-gene ROC analysis; validation set for single-gene expression levels in normal tissues vs. AS tissues	32	32
GSE159677	Single-cell transcriptomic analysis of AS	3	3

### 2.2 Analyzing CRC and AS gene expression data by weighted gene co-expression network analysis

We used the WGCNA package (v.1.72-5) to analyze two cohorts: the TCGA-COADREAD patient cohort (CRC as phenotypic trait) and the GSE100927 dataset (AS as phenotypic trait). The analysis process started with sample quality control using the goodSamplesGenes function, followed by hierarchical clustering to remove abnormal samples. Subsequently, we determined the optimal soft-thresholding power to achieve a scale-free network structure, maintaining minimum mean connectivity while aiming for a scale-free topology fit index (*R*²) exceeding 0.85. Thereafter, we constructed a hierarchical clustering dendrogram based on gene expression patterns and quantified the association matrices between module eigengenes and clinical traits. Different module size thresholds were applied - 30 genes for AS studies and 60 genes for CRC investigations - in accordance with the specifications of the dynamic tree-cutting algorithm. The resulting interrelationships were visualized through a correlation heatmap. The critical modules showing the strongest associations with CRC and AS (absolute correlation coefficient ≥ 0.3, *p*-value < 0.05) were prioritized for subsequent analysis.

After identifying modules associated with CRC and AS via WGCNA, we adjusted the screening criteria as follows: Modules with the smallest P-value, significantly higher correlation between the disease/tumor group and the phenotypic trait than that in the normal group, and |*r*| ≥ 0.3 in the disease group were selected as the core pathogenic gene modules. Specifically, in the CRC study, we selected the yellow module with a correlation coefficient |*r*| = 0.74 with the CRC phenotype (significantly higher than the correlation of the corresponding module in the normal group). In the AS study, we selected the turquoise module with a correlation coefficient |*r*| = 0.69 with the AS phenotype (significantly higher than the correlation of the corresponding module in the normal group) as the core pathogenic module for each study to conduct subsequent in-depth analysis.

### 2.3 Identification and analysis of differentially expressed genes in CRC and gene enrichment analysis

Using the limma package (v.3.58.1), the differential expression analysis was conducted. Expression profiles from normal and tumor specimens were integrated via a constructed design matrix. Genes showing differential expression (DEGs) were identified by linear modeling combined with empirical Bayes methods, with thresholds of |log2FC| ≥ 1 and an adjusted *p*-value < 0.05 for statistical significance. Based on the logFC values of DEGs, the DEGs were classified into upregulated and downregulated groups respectively.

Subsequently, the “overlapping genes” were clearly defined as the intersection of three gene sets, namely: the yellow module genes (screened via WGCNA, which exhibited the strongest association with CRC), the turquoise module genes (screened via WGCNA, which showed the most significant correlation with AS), and the DEGs identified in CRC. This defined overlapping gene set was then systematically analyzed to explore their shared molecular characteristics underlying both diseases. Thereafter, the clusterProfiler package (v.4.14.3) was employed for functional annotation of these overlapping genes, and enrichment analyses were performed for Kyoto Encyclopedia of Genes and Genomes (KEGG) pathways and Gene Ontology (GO) terms, with a statistical significance threshold set at *p* < 0.05.

### 2.4 CRC risk model: score computation, stratification & validation for clinical outcome predictions

We used the survival package (v.3.5-7) to perform univariate Cox regression analysis, integrating the expression profiles of “overlapping genes” in CRC patients with clinical survival parameters (survival time and death-based outcomes). The goal was to screen genes with statistical significance (*p* < 0.05). Subsequently, the least absolute shrinkage and selection operator (LASSO) regression, implemented in the glmnet package (v.4.1-8), was applied to analyze these filtered genes. Cross-validation was used to determine the optimal regularization parameter (*λ*), specifically adopting a 10-fold cross-validation strategy and selecting the optimal *λ* value based on the criterion of minimum cross-validation deviance (corresponding to the lambda.min parameter output by the glmnet package). Feature genes with non-zero coefficients at this critical *λ* value were incorporated into the common diagnostic genes and the CRC risk model.

Further analyses were conducted based on the constructed risk model. Individual risk scores were calculated by multiplying the gene expression value of each sample by its corresponding LASSO-derived weight. After integrating survival data, the optimal cutoff value was determined using the median method of risk scores (for the training set) and SRplot (17) (for the validation set) to establish the best stratification threshold for grouping. Sangerbox was used to evaluate the relationships between different risk scores and patients’ follow-up time, events, as well as expression changes of each gene (18).

### 2.5 Gene set enrichment analysis within the risk group

Gene expression profiles from TCGA-COADREAD were used to carry out Gene Set Enrichment Analysis (GSEA) for comparing the two risk groups. The clusterProfiler package (v.4.14.3) enabled the examination of both KEGG pathways and Hallmark gene sets. Differently expressed genes (DEGs) differentiating the two risk groups were sorted based on their logFC values, and the GSEA methodology was used to determine the pathway enrichment scores. The visualization of results involved generating enrichment plots that depicted significant pathway activation patterns.

### 2.6 Analysis of immune infiltration and checkpoints in the risk group

The infiltration patterns of immune cells were evaluated through the CIBERSORT algorithm implemented in IOBR package (v 0.99.0), revealing distinct immunological profiles among different risk stratifications. Statistical comparisons of immune cell proportions between the high-risk and low-risk cohorts were performed using the Wilcoxon test, with boxplot visualizations indicating statistically significant distribution variances. Furthermore, a comprehensive assessment of 34 critical immune checkpoint molecules (19) across risk categories was carried out, comparing their differential expression patterns and exploring potential implications for immunotherapeutic interventions. This analysis also extended to examining the functional correlations between checkpoint expression levels and treatment responsiveness, highlighting their relevance for developing personalized therapeutic strategies.

### 2.7 Drug sensitivity analysis of immune checkpoint inhibitors in the risk group

Using the oncoPredict package (version v1.2) in R, we calculated the half-maximal inhibitory concentration (IC50) values of 198 immune checkpoint inhibitor (ICI) drugs retrieved from the Genomics of Drug Sensitivity in Cancer (GDSC, URL: https://www.cancerrxgene.org/) database for tumor samples, based on the risk stratification results of the TCGA-COADREAD dataset. Differences in IC_50_ values of these 198 drugs between the high- and low-risk groups were compared using the Wilcoxon rank-sum test.

### 2.8 Diagnostic value and expression analysis of prognostic genes

The diagnostic potential of the candidate diagnostic genes was evaluated through Receiver Operating Characteristic (ROC) curve analysis, which was generated via the pROC package (v1.18.5). We explored the expression patterns in colorectal tissues by making use of both TCGA-COADREAD and GSE87211 cohorts to compare the transcriptional profiles between healthy and tumor samples. Furthermore, the differential expression profiles were examined in atherosclerotic specimens through the analysis of GSE100927and GSE43292 datasets, contrasting the vascular tissue samples from normal controls with those having atherosclerotic lesions.

### 2.9 Spatial transcriptomic data processing and visualization

In this study, spatial transcriptomic data from three CRC samples were obtained from the publicly available dataset GSE225857 and processed using Seurat v4. H&E-stained tissue images were visualized using the SpatialDimPlot function, and tumor and normal regions were manually annotated based on tissue morphology. For data quality control and normalization, the SCTransform function was applied with the glmGamPoi method to normalize spatial transcriptomic counts. Transcript count distributions across spatial spots were assessed using VlnPlot and SpatialFeaturePlot. Subsequently, six prognostic model genes (*CDC25C*, *HMMR*, *KPNA2*, *PRR11*, *PALB2*, and *TKT*) were selected for spatial expression analysis. Their spatial expression patterns across tissue sections from different patients were visualized using SpatialFeaturePlot.

### 2.10 Single cell RNA-seq data processing and analysis

This study employed the Seurat R package (v4.4.0) to process and analyze two single-cell RNA sequencing datasets: GSE132465 (CRC) and GSE159677 (AS). Initial quality control was performed separately on both datasets. For GSE132465, cells with >10% mitochondrial gene expression, fewer than 200 or more than 6000 detected genes, or fewer than 1000 total transcripts were excluded. For GSE159677, cells with >20% mitochondrial gene expression, fewer than 200 or more than 6000 detected genes, or more than 30,000 transcripts were removed. Additionally, genes expressed in fewer than three cells were filtered out. After quality control, a total of 54,541 cells from GSE132465 and 49,064 cells from GSE159677 were retained for downstream analysis. Subsequent analysis followed standard Seurat workflows. For both datasets, normalization (NormalizeData), identification of highly variable features (FindVariableFeatures), and scaling (ScaleData) were performed. Principal component analysis (PCA) was conducted using the top 30 principal components. A shared nearest neighbor (SNN) graph was constructed for clustering, with resolution parameters set to 0.1 for CRC and 0.2 for AS. Dimensionality reduction for visualization was performed using Uniform Manifold Approximation and Projection (UMAP). Cell type annotation was based on canonical marker gene expression. For the CRC dataset, annotated cell types included T/NK cells (*CD3D*, *CD3E*, *TRAC*), B cells (*MS4A1*, *CD79A*, *CD79B*), plasma cells (*MZB1*, *TNFRSF17*), myeloid cells (*CD14*, *CD68*, *LYZ*), plasmacytoid dendritic cells (*LILRA4*), mast cells (*TPSAB1*), vascular endothelial cells (*PECAM1*, *VWF*, *CLDN5*), stromal fibroblasts (*COL1A2*, *COL3A1*, *ACTA2*), and epithelial cells (*EPCAM*, *CD24*). For the AS dataset, cell types were annotated as macrophages (*AIF1*, *CD14*, *CD68*), vascular endothelial cells (*VWF*, *PECAM1*, *ECSCR*), vascular smooth muscle cells (*CALD1*, *MYL9*, *TAGLN*), natural killer cells (*NKG7*, *XCL1*, *CTSW*), T cells (*CD2*, *TRAC*, *CD69*), and B cells (*CD79A*, *MS4A1*, *IGKC*).

### 2.11 Prediction of transcription factors for six biomarkers and construction-visualization of mRNA-TF networks

Based on six biomarkers (*PRR11*, *HMMR*, *CDC25C*, *KPNA2*, *TKT*, and *PALB2*), we predicted the transcription factors (TFs) corresponding to the mRNA of each biomarker via the ChEA3 database (URL: https://maayanlab.cloud/chea3/), constructed mRNA-TF networks, and performed network visualization using Cytoscape software.

### 2.12 Biomarker interacting gene mining, functional enrichment and network construction via GeneMANIA

Using the GeneMANIA database (URL: http://genemania.org/), we performed analysis and network construction for the biomarkers, displayed genes with strong interaction relationships with the biomarkers, and simultaneously presented the top 7 functions ranked by false discovery rate (FDR) values in ascending order.

### 2.13 Clinical samples and quantitative real-time polymerase chain reaction

At Sun Yat-sen Memorial Hospital affiliated with Sun Yat-sen University, 23 paired specimens (primary tumor lesions and adjacent normal tissues) were collected from CRC patients. This study was approved by the hospital’s Ethics Committee (approval no. SYSKY-2025-426-01), and all participants provided written informed consent.

Total RNA was isolated using Trizol reagent (Vazyme, Nanjing, China), followed by cDNA synthesis via reverse transcription with a Vazyme kit. Quantitative real-time PCR (qRT-PCR) was performed with ChamQ Universal SYBR qPCR Master Mix (Vazyme), and relative gene expression was calculated using the 2^-^ΔΔCt method. Outliers in the qRT-PCR data were removed by the interquartile range (IQR) method.

Gene expression differences between paired tumor and normal tissues were analyzed using the *Wilcoxon signed-rank* test (paired nonparametric test). Detailed qRT-PCR primer sequences are available in **Supplementary Table S2**.

### 2.14 Western blot detection of HMMR and PALB2 protein expression

Six pairs of postoperative pathological specimens (approximately 0.3 cm in diameter) from CRC patients were collected at Sun Yat-sen Memorial Hospital, Sun Yat-sen University. CRC tissues and adjacent normal tissues were ground in a cryo-grinder, then lysed with 200 μL of high-strength RIPA lysis buffer (Fdbio Science, Zhejiang, China) containing 1% protease inhibitor, 1% protein phosphatase inhibitor, and 1% phenylmethylsulfonyl fluoride (PMSF, Fdbio Science). After thorough mixing, the lysates were centrifuged at 12,000 r/min for 15 min at 4°C, and the supernatants were collected. Total protein concentration was quantified using a BCA protein assay kit (Beyotime Biotechnology, Jiangsu, China), and samples were normalized to equal concentrations. Protein samples were mixed with 5× SDS loading buffer (Fdbio Science, Zhejiang, China), denatured by boiling at 95°C for 10 min, and separated by 10% SDS-PAGE under constant voltage (120 V). The gels used were 10-well gels, with Well 1 and Well 10 loaded with Marker from Yazyme (Cat. No.: WJ103). Proteins were wet-transferred to a PVDF membrane (Roche, Cat. No. 03010040001, pore size: 0.2 μm, Switzerland) using transfer buffer containing 25 mM Tris-glycine and 20% methanol at a constant current of 150 mA for 90 min with ice-bath cooling. The membrane was blocked with QuickBlock™ Blocking Buffer for Western Blot (Beyotime Biotechnology, Jiangsu, China) at room temperature for 1 h, washed with TBST, and incubated overnight at 4°C with primary antibodies: HMMR (1:2000, 15820-1-AP, Proteintech), PALB2 (1:2000, 14340-1-AP, Proteintech), and β-actin (1:10,000, 66009-1-Ig, Proteintech). After washing, the membrane was incubated with HRP-conjugated secondary antibodies: goat anti-mouse IgG (1:10,000, SA00001-1, Proteintech) and goat anti-rabbit IgG (1:10,000, SA00001-2, Proteintech) at room temperature for 1 h, followed by three TBST washes (10 min each). Specific protein bands were visualized using FDbio-Femto Ecl chemiluminescent detection reagent (Fdbio Science, Zhejiang, China) and imaged with a Touch Imager™ system, and relative expression levels were analyzed by ImageJ software with β-actin as the internal reference.

### 2.15 IHC staining of HMMR, PALB2, and PRR11 in CRC and AS pathological slides

Seven pairs of specimens from patients with CRC (paired with adjacent normal tissues as controls) and atherosclerotic patients (including 7 artery atherosclerotic tissues and 5 normal renal artery tissues) were collected at Sun Yat-sen Memorial Hospital, Sun Yat-sen University. For the paraffin sections, dewaxing and rehydration were performed by sequential immersion in xylene I and II for 15 min each, followed by a gradient series of absolute ethanol to 50% ethanol. Antigen retrieval was then carried out using a pH 9.0 Tris-EDTA retrieval solution (ZGSB-BIO, BEIJING, ZLI-9069) via microwave treatment for 10 min. Endogenous peroxidase activity was blocked with 3% hydrogen peroxide for 15 min at room temperature. After blocking non-specific binding sites with 5% BSA at 37°C for 1 h, primary antibodies [HMMR (15820-1-AP, Proteintech), PALB2 (14340-1-AP, Proteintech), and PRR11 (BD-PE4086, Biodragon)], all diluted at 1:50, were applied and incubated overnight at 4°C. Following 1×PBS washes, the sections were sequentially incubated with an enhancement solution for 20 min at room temperature, secondary antibodies (PV-6001/PV-6002, ZGSB-BIO, BEIJING), and visualized using DAB chromogenic solution (ZGSB-BIO, ZLI-9019) under a microscope. After counterstaining with hematoxylin solution (JLM-111, Jieli, Guangzhou), the sections were dehydrated through a graded ethanol series, cleared with xylene, and mounted with neutral gum. Immunohistochemical results were scored using the product of “staining intensity (scored 0-3, from negative to strongly positive)” and “staining area (scored 0-4, corresponding to 0-100% positive cell proportion)”.

### 2.16 Statistical analysis

Statistical analyses were carried out using R software (v4.2.1). For comparison analyses between groups, the nonparametric Wilcoxon rank-sum test was employed; the statistical significance levels are as follows: *p < 0.05, **p < 0.01, ***p < 0.001, and ****p < 0.0001.

## 3 Results

### 3.1 Identification and enrichment analysis of the shared pathogenic genes in CRC and AS

**Figure 2:**
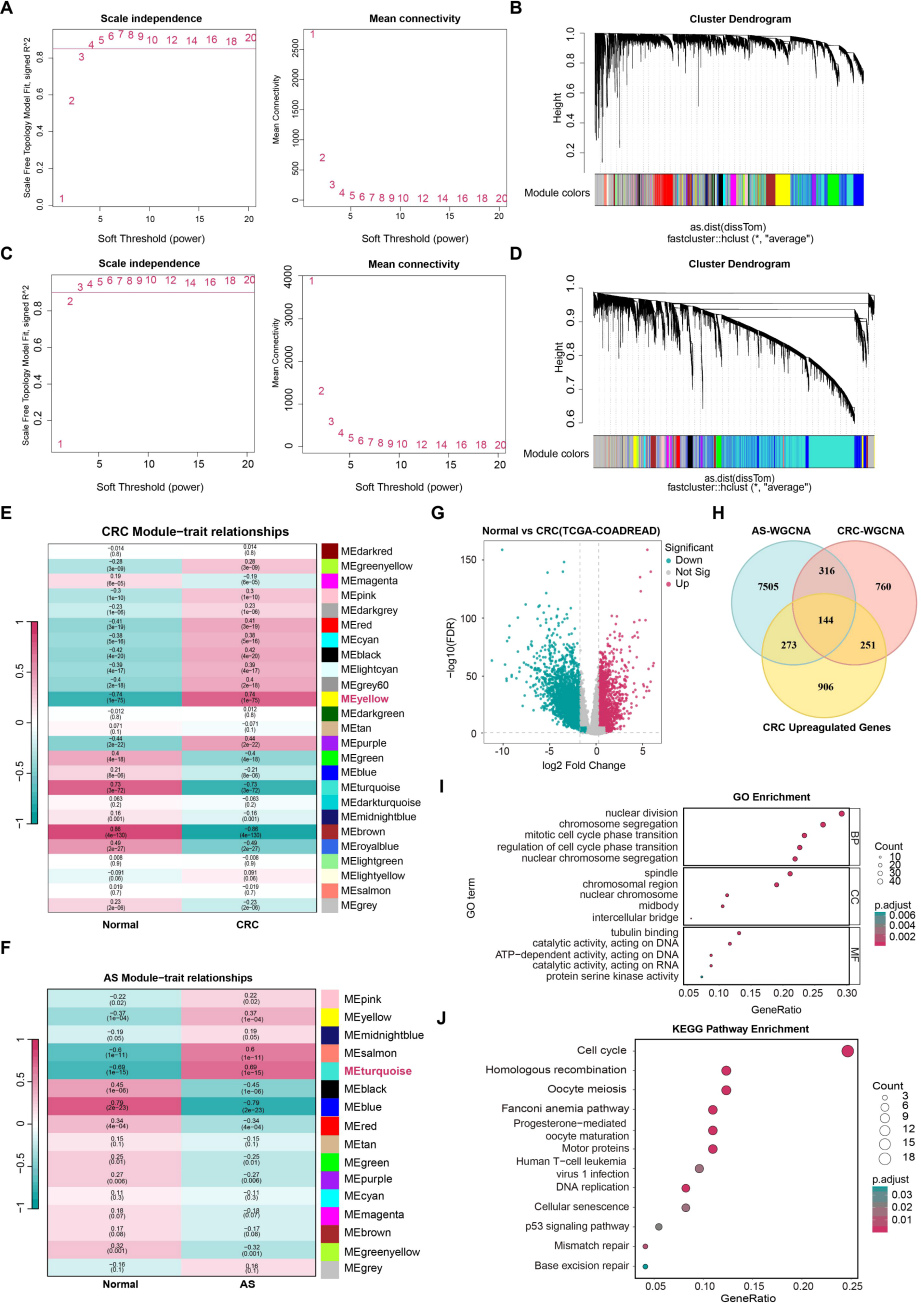
Intersection and functional enrichment of WGCNA module genes in CRC and AS, and differential genes in CRC. **(A, C)** Soft threshold power and mean connectivity plots for determining the optimal soft-thresholding powers in CRC and AS datasets. **(B, D)** Cluster dendrograms showing the hierarchical clustering of genes in CRC and AS. **(E, F)** Module clustering results, with colors representing different modules. Modules with the highest positive correlations were identified for further analysis. **(G)** Volcano plot showing the expression patterns of differentially expressed genes (DEGs). **(H)** Venn diagram illustrating the intersection of DEGs with module genes identified in WGCNA for CRC and AS. **(I, J)** GO and KEGG enrichment analyses highlighting key biological functions and pathways, including cell cycle regulation and DNA repair, enriched among shared genes.

We constructed gene co-expression modules for CRC and AS using the WGCNA, aiming to screen core gene modules associated with these diseases. For CRC analysis based on the TCGA-COADREAD dataset, a soft-thresholding power of 4 was determined (**Figure 2A**), and a total of 25 co-expression modules were identified (**Figure 2B**). For AS analysis based on the GSE100927 dataset, a soft-thresholding power of 4 was also selected (**Figure 2C**), leading to the identification of 16 co-expression modules (**Figure 2D**). Among these, the yellow module showed the strongest association with the disease phenotype, containing 1,471 genes in CRC (**Figure 2E**). The turquoise module exhibited the most significant correlation with AS and included 8,238 genes (**Figure 2F**).

Differential expression analysis of the CRC dataset identified 1,574 genes that were upregulated in tumor tissues compared to normal tissues (**Figure 2G**). Further intersection analysis of genes from the AS turquoise module (8,238 genes), CRC yellow module (1,471 genes), and CRC upregulated genes (1,574 genes) revealed 144 shared genes (**Figure 2H**). Functional enrichment analysis of these 144 shared genes yielded the following results: GO enrichment analysis revealed that they were predominantly involved in biological processes like nuclear division, organelle fission, and chromosome segregation (**Figure 2I**), with a notable enrichment in chromosome segregation and cell division. KEGG enrichment analysis showed that these genes were significantly enriched in pathways including the cell cycle, homologous recombination, Fanconi anemia pathway, DNA replication, and mismatch repair (**Figure 2J**).

### 3.2 Machine learning-based screening of shared genes for CRC and AS: CRC risk model construction, risk stratification, and prognostic validation

**Figure 3:**
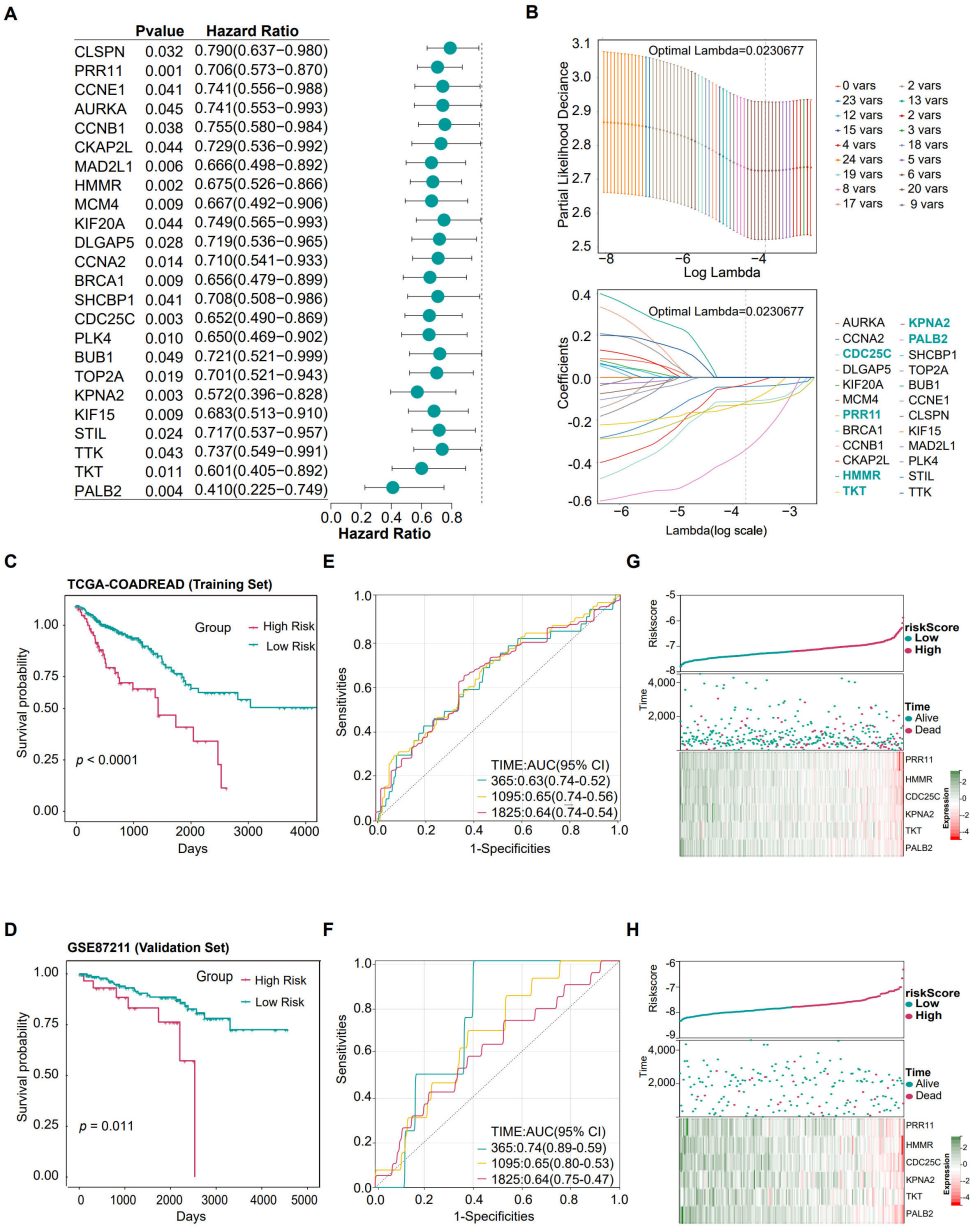
Gene screening, construction, performance validation and risk correlation analysis of the CRC risk model. **(A)** Univariate Cox regression analysis identifying genes associated with CRC prognosis. **(B)** LASSO regression analysis refining prognostic genes. **(C, D)** Kaplan-Meier survival curves showing significant differences in overall survival (OS) between low-risk and high-risk groups. **(C)** represents the training set TCGA-COADREAD; **(D)** represents the validation set GSE87211 dataset. **(E, F)** ROC curves demonstrating the predictive accuracy of the risk model, with area under the curve (AUC) values calculated for 1–5 years. **(E)** Training set TCGA-COADREAD; **(F)** Validation set GSE87211. **(G, H)** Relationships between risk scores, patient follow-up time, events, and gene expression changes in the training set TCGA-COADREAD and validation set GSE87211. **(G)** Training set TCGA-COADREAD; **(H)** Validation set GSE87211.

Based on the 144 genes commonly expressed in both CRC and AS identified in the previous step, we performed univariate Cox regression analysis on the TCGA-COADREAD dataset and identified 24 genes significantly associated with prognosis (**Figure 3A**). These genes exhibited a protective role in CRC progression. Further analysis using the LASSO regression algorithm identified six robust prognostic genes: Cell Division Cycle 25C (*CDC25C*), Hyaluronan-Mediated Motility Receptor (*HMMR*), Karyopherin Subunit Alpha 2 (*KPNA2*), Partner and Localizer of BRCA2 (*PALB2*), Proline-Rich Protein 11 (*PRR11*), and Transketolase (*TKT*) (**Figure 3B**). Survival curves comparing high- and low-expression groups for these six genes are shown in **Supplementary Figure S1**.

The CRC risk score model was constructed using these six genes: Risk score = −0.127356137×*PRR11* expression − 0.042220722×*HMMR* expression − 0.111721191×*CDC25C* expression − 0.03272567×*KPNA2* expression − 0.117397 ×*TKT* expression − 0.358569689×*PALB2* expression.

In the TCGA-COADREAD training set, Kaplan-Meier survival analysis (**Figure 3C**) showed that the overall survival rate of CRC patients in the high-risk group was significantly lower than that in the low-risk group (*p* < 0.05), clearly indicating a statistical association between elevated risk scores and poor prognosis in patients. Further evaluation of the prognostic predictive efficacy of the risk model using ROC curve (**Figure 3D**) revealed that the AUC values of the model for predicting 1-year, 2-year, 3-year, 4-year, and 5-year survival outcomes were 0.64, 0.67, 0.67, 0.65, and 0.63, respectively, demonstrating that it has moderate or higher predictive accuracy in the training set. The triptych analysis (**Figure 3E**) verified the clinical significance of the risk score from multiple dimensions: as the risk score increased, the proportion of deceased samples gradually rose, and the expression levels of the 6 diagnostic biomarkers all decreased.

Similar findings were observed in the validation set (GSE87211). The Kaplan-Meier curve (**Figure 3F**) replicated the key finding of the training set, that is, the survival rate of patients in the high-risk group was significantly lower than that in the low-risk group (*p* < 0.05), confirming the stability and reliability of risk stratification. ROC curve analysis (**Figure 3G**) showed that the AUC values of the model for predicting 1-year, 2-year, 3-year, 4-year, and 5-year survival outcomes in the validation set were 0.75, 0.68, 0.67, 0.57, and 0.60, respectively. Among them, the predictive efficacy for 1-year survival rate was improved compared with the training set, which further supports the clinical application value of the model. The triptych results (**Figure 3H**) reproduced the above-mentioned association pattern in the validation set: elevated risk scores were accompanied by an increase in the number of deceased samples, and the expression abundance of the 6 genes decreased.

### 3.3 GSEA enrichment analysis, immune infiltration, immune checkpoint analysis and drug sensitivity analysis of ICIs in CRC high- and low-risk group

**Figure 4:**
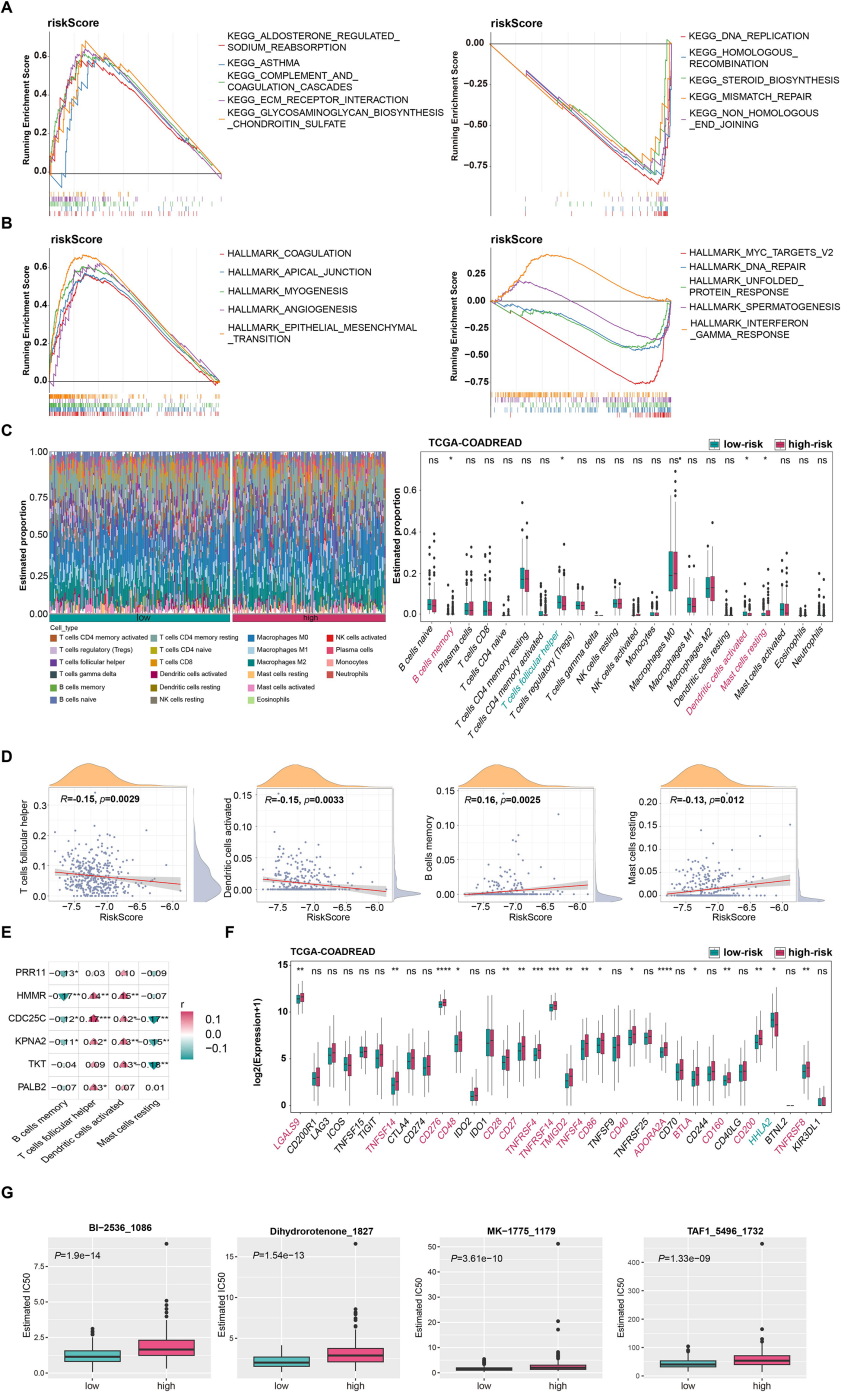
GSEA enrichment analysis, immune infiltration characteristics, and immune checkpoint differential analysis in high- and low-risk groups of the CRC model. **(A)** GSEA enrichment analysis of KEGG pathways in high- and low-risk groups. **(B)** GSEA enrichment analysis of Hallmark gene sets in high- and low-risk groups. **(C)** Relative infiltration abundance of 22 immune cell types in high- and low-risk groups (left); Comparison of immune cell infiltration levels between high- and low-risk groups (right). **(D)** Correlation scatter plot between risk scores and infiltration levels of significantly differential immune cells. **(E)** Correlation analysis between 6 genes in the model and differential immune cells. **(F)** Differential expression of immune checkpoints between high- and low-risk group. **(G)** Boxplots of the four chemotherapeutic drugs with the smallest *p*-values for IC50 differences between high- and low-risk groups.

KEGG enrichment analysis of the high- and low-risk groups based on the CRC risk model showed that the low-risk group was significantly enriched in pathways such as aldosterone-regulated sodium reabsorption, complement and coagulation cascades, and extracellular matrix receptor interaction. These pathways are mainly involved in extracellular matrix remodeling, inflammatory response, and metabolic regulation, suggesting that their biological functions focus on metabolism and extracellular matrix regulation. The high-risk group was centered on pathways such as DNA replication, homologous recombination, and mismatch repair (**Figure 4A**).

Hallmark enrichment analysis further indicated that the low-risk group was significantly enriched in processes such as coagulation, angiogenesis, and epithelial-mesenchymal transition, participating in angiogenesis, cell adhesion, and tissue remodeling. The high-risk group was enriched in pathways including MYC target genes V2, DNA repair, and gamma interferon response, which are involved in DNA repair, cellular stress, and immune response, suggesting abnormalities in coping with cellular damage and immune regulation (**Figure 4B**).

Assessment of immune cell infiltration using CIBERSORT revealed significant differences in the proportions of immune cells between the high- and low-risk groups (**Figure 4C**). The low-risk group had more abundant follicular helper T cells and activated dendritic cells, while the high-risk group was mainly composed of memory B cells and resting mast cells (**Figure 4D**). **Figure 4E** presents the results of correlation analysis between the expression of *PRR11*, *HMMR*, *CDC25C*, *KPNA2*, *TKT*, and *PALB2* genes and the infiltration levels of memory B cells, follicular helper T cells, activated dendritic cells, and resting mast cells in the context of high-risk and low-risk groups: 1) All six genes were negatively correlated with memory B cells, among which the differences in the associations of *PRR11*, *HMMR*, *CDC25C*, and *KPNA2* with memory B cells were statistically significant; 2) *HMMR*, *CDC25C*, *KPNA2*, and *PALB2* were also positively correlated with follicular helper T cells and activated dendritic cells; however, the differences in the positive correlations of *PRR11* with these two types of cells were not statistically significant. Meanwhile, the positive correlations of *TKT* with follicular helper T cells and of *PALB2* with activated dendritic cells were not statistically significant, respectively. 3) *CDC25C*, *KPNA2*, and *TKT* were negatively correlated with resting mast cells, and the differences were statistically significant. Immune checkpoint analysis revealed that multiple indicators had statistical significance. Among them, the most statistically significant ones were *CD276*, *ADORA2A*, *TNFRSF4*, and *TNFRSF14*, which were significantly upregulated in the high-risk group (*p* < 0.0001) (**Figure 4F**).

Based on drug information from the GDSC database encompassing 198 chemotherapeutic/targeted agents and risk stratification of the TCGA-COADREAD cohort, we assessed differences in drug sensitivity between high- and low-risk groups. Wilcoxon rank-sum tests revealed that 86 agents exhibited significantly different IC50 values between the two risk groups (*p* < 0.05), with the high-risk group showing consistently higher IC50 values for these compounds (**Supplementary Figure S2**). The four most significantly differential drugs (BI-2536, Dihydrorotenone, MK-1775 , and TAF1) were selected for visualization, as shown in **Figure 4G**.

### 3.4 Diagnostic value and transcriptional expression levels of six hub genes in CRC and AS

**Figure 5:**
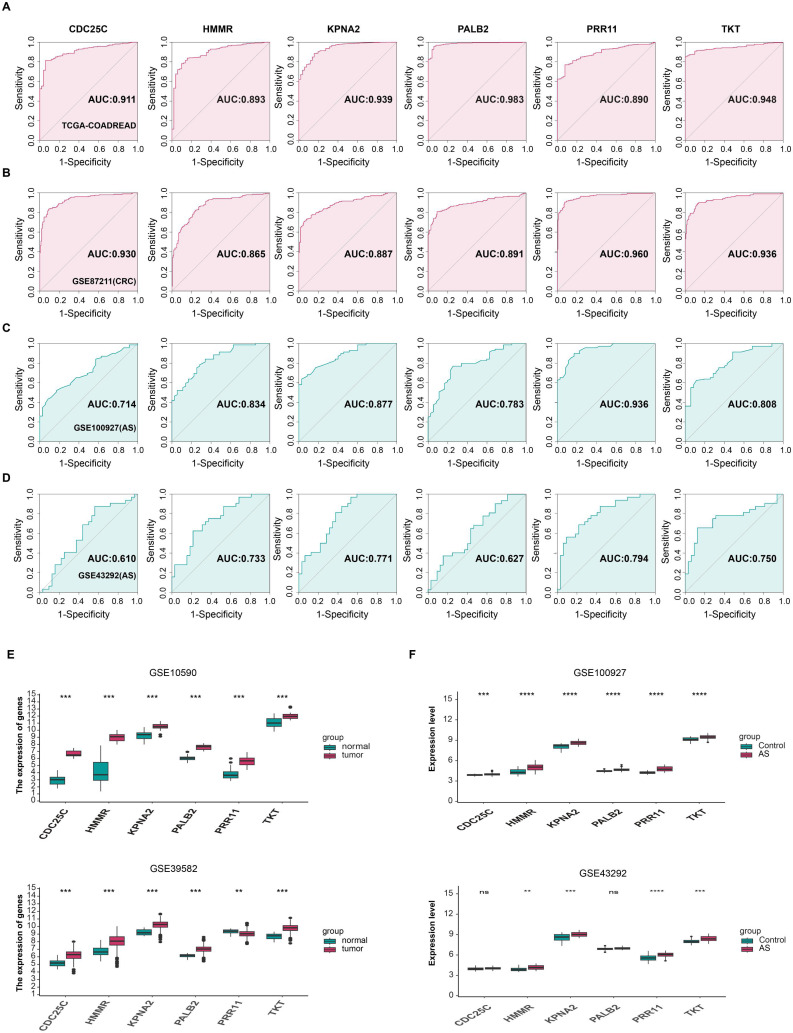
Validation of diagnostic efficacy and multi-dataset differential expression analysis of 6 hub genes in CRC and AS. **(A-D)** ROC curves verifying the diagnostic reliability of 6 hub genes: **(A)** CRC training set; **(B)** CRC validation set; **(C)** AS training set; **(D)** AS validation set. **(E, F)** Differential expression analysis of 6 hub genes in two CRC datasets (normal vs tumor tissues) and two AS datasets (normal vs lesioned tissues). **p < 0.01, ***p <0.001, ****p < 0.0001. ns indicates no statistical significance.

Building on the preliminary exploration of the expression patterns of these candidate diagnostic genes, we further validated their diagnostic efficacy through receiver operating characteristic (ROC) curve analysis in training and validation sets. We explored the expression patterns of these six genes in the CRC training set (TCGA-COADREAD), CRC validation set (GSE87211), AS training set (GSE100927), and AS validation set (GSE43292) (**Figures 5A-D**); the results showed that *PRR11* exhibited expression levels of 0.89 and 0.96 in the CRC training set and validation set, respectively, and 0.936 and 0.794 in the AS training set and validation set, respectively. *CDC25C* showed expression levels of 0.911 and 0.93 in the CRC training set and validation set, respectively, and 0.714 and 0.61 in the AS training set and validation set, respectively. *HMMR* had expression levels of 0.893 and 0.865 in the CRC training set and validation set, respectively, and 0.834 and 0.733 in the AS training set and validation set, respectively. *KPNA2* displayed expression levels of 0.939 and 0.887 in the CRC training set and validation set, respectively, and 0.877 and 0.771 in the AS training set and validation set, respectively. *PALB2* presented expression levels of 0.983 and 0.891 in the CRC training set and validation set, respectively, and 0.783 and 0.627 in the AS training set and validation set, respectively. *TKT* demonstrated expression levels of 0.948 and 0.936 in the CRC training set and validation set, respectively, and 0.808 and 0.75 in the AS training set and validation set, respectively.

Subsequently, we analyzed the expression levels of these six genes in CRC using two CRC transcriptome datasets, GSE10590 and GSE39582. In the GSE10590 dataset, all six genes exhibited higher expression levels in tumor tissues than in normal tissues. In GSE39582, however, the expression level of *PRR11* was lower in tumor tissues than in normal tissues (**Figure 5E**). In AS transcriptome datasets, all six genes also showed higher expression levels in lesioned tissues compared to control tissues in the GSE100927 (training set). In the GSE43292 (validation set), although the expression levels of these genes in lesioned tissues were also higher than those in control tissues, the differences were not statistically significant for *CDC25C* and *PALB2* (**Figure 5F**).

### 3.5 Expression profiles of dual-disease prognostic core genes in single-cell and spatial transcriptomics data

**Figure 6:**
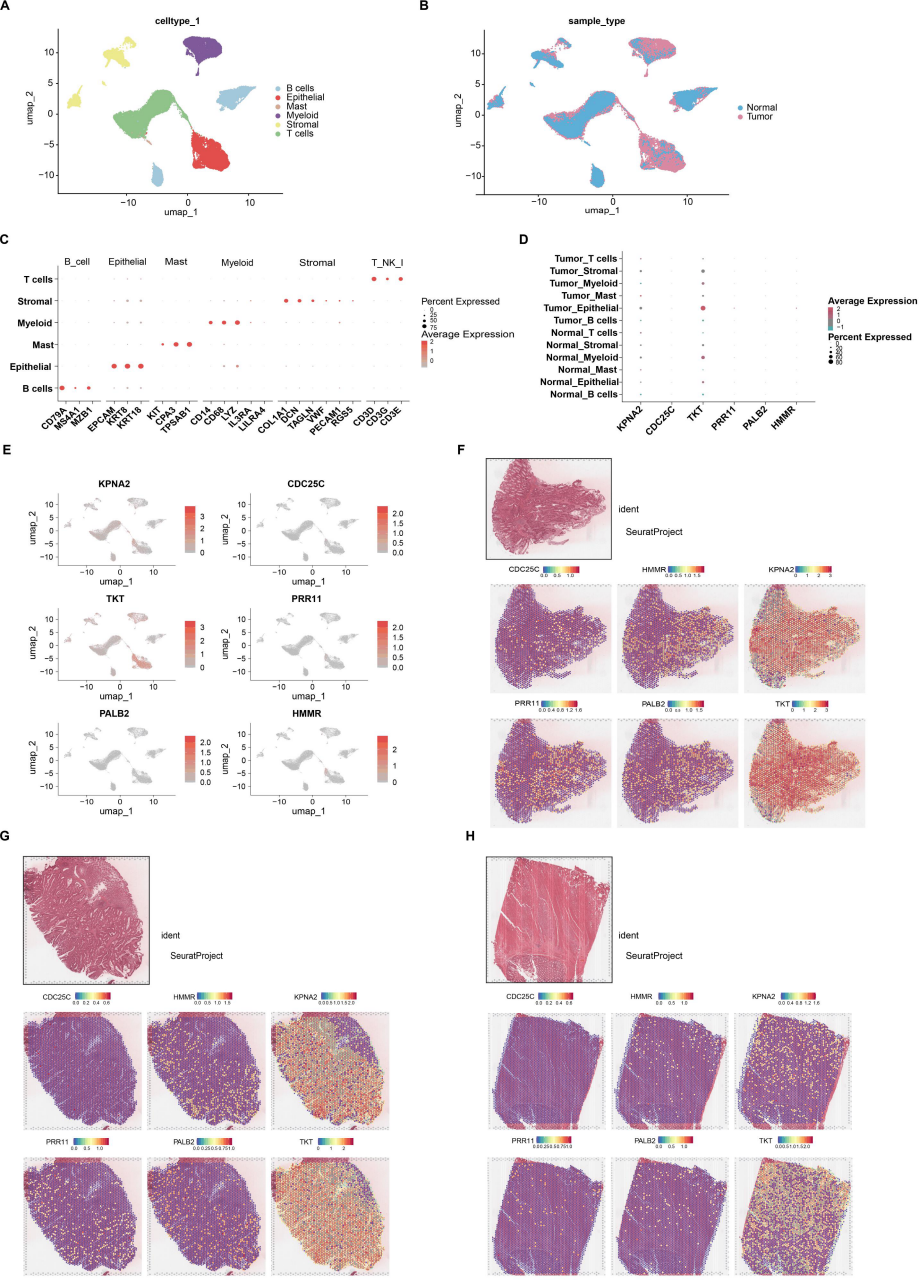
Hub genes analysis based on single-cell and spatial transcriptomics in CRC. **(A)** Uniform Manifold Approximation and Projection (UMAP) plots of high-quality cells (n = 54,541) from all enrolled samples (n = 33) visualizing celltype clusters. (B, B cell; Epi, Epithelial cell; Mast, Mast cell; Myeloid, Myeloid cell; Stromal, Stromal cell; T, T cell). **(B)** UMAP plot of tumor tissue and normal tissue distribution. **(C)** Expression levels of marker genes across six major cell types. **(D)** UMAP visualization of expression levels of six key core genes. **(E)** The dot plot illustrates the expression levels of six key core genes across different cell populations in colorectal cancer tissue and adjacent normal tissue. **(F-H)** Spatial plots showing the spatial expression pattern of six key core genes using GSE225857 dataset.

To investigate the expression patterns of six hub genes (*KPNA2*, *CDC25C*, *TKT*, *PRR11*, *PALB2,* and *HMMR*) identified from a dual-disease prognostic model, we analyzed single-cell transcriptomic data comprising 54,541 high-quality cells from 33 CRC samples. UMAP clustering showed major cell type distributions (**Figure 6A**), and cells were further classified based on tissue origin into tumor and adjacent normal tissues (**Figure 6B**). Marker gene expression was used to validate the accuracy of cell-type annotations (**Figure 6C**). UMAP plots visualized the expression patterns of the six core genes across different cell populations (**Figure 6D**). Further dot plot analysis revealed that *TKT* and *KPNA2* were relatively highly expressed in epithelial and myeloid cells, with *TKT* showing notably higher expression in tumor epithelial cells compared to normal epithelial cells, suggesting its potential involvement in tumorigenesis or progression. In contrast, the other genes exhibited generally low expression levels across most cell types (**Figure 6E**).

To further investigate the spatial expression characteristics of the key genes in our prognostic model, we analyzed six hub genes (*KPNA2*, *CDC25C*, *TKT*, *PRR11*, *PALB2*, and *HMMR*) based on the publicly available spatial transcriptomics dataset GSE225857 from CRC patients. Hematoxylin and eosin (H&E) staining was used to annotate tumor and adjacent normal regions, providing morphological context for spatial expression interpretation. The results revealed distinct spatial heterogeneity in the expression of these genes across the tissue section. *KPNA2* and *TKT* were broadly and highly expressed within tumor regions, suggesting their potential involvement in tumor proliferation or metabolic activity. *CDC25C*, *HMMR*, and *PRR11* exhibited focal areas of increased expression, indicating spatial variation in functional states within the tumor. *PALB2* showed a more uniform distribution without obvious tumor-specific enrichment. These findings highlight the spatial expression patterns of prognostic model genes within the tumor microenvironment and support their potential roles in CRC progression (**Figures 6F-H**).

**Figure 7:**
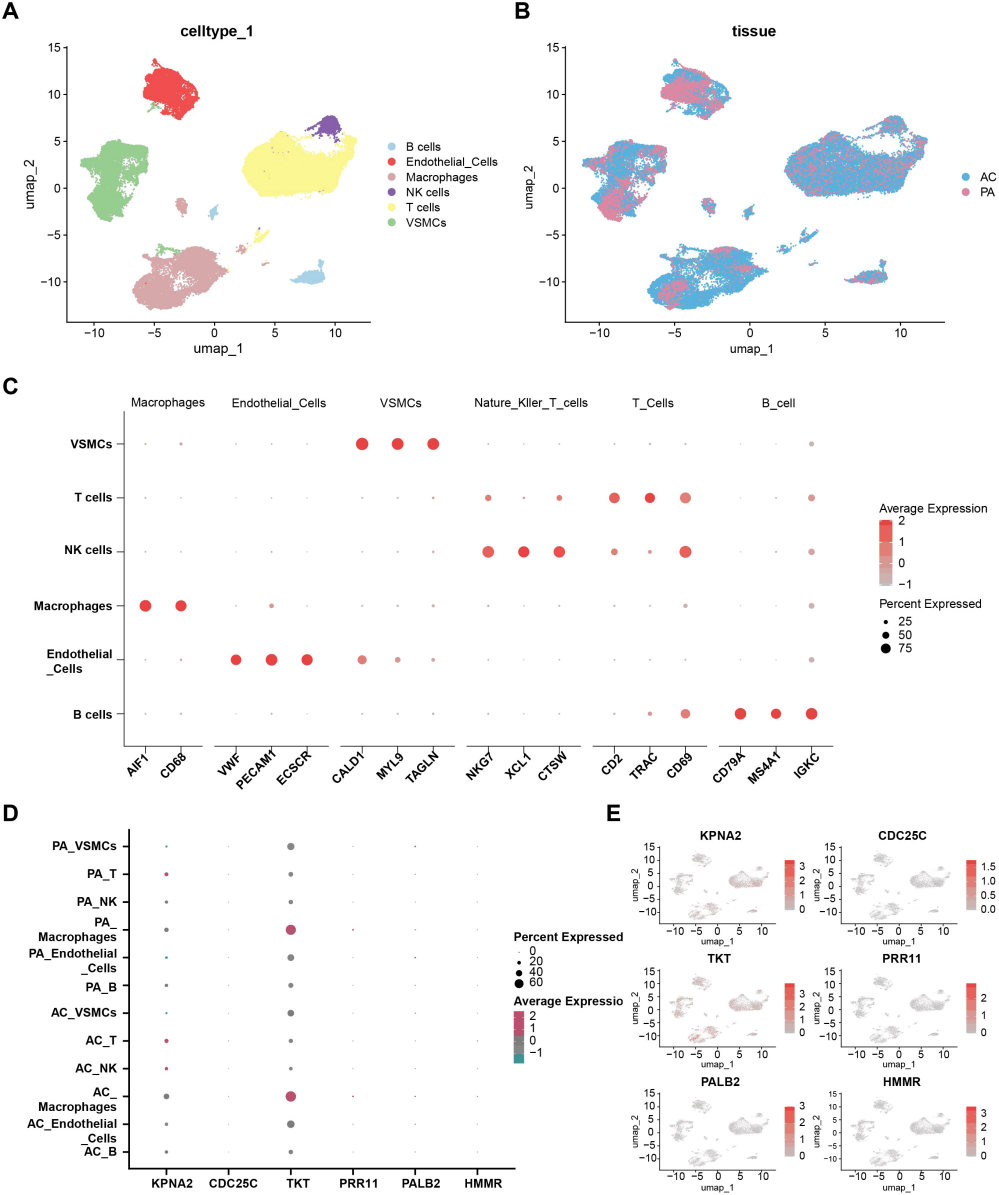
Prognosis-related genes analysis based on single-cell sequencing in atherosclerotic plaque. **(A)** Uniform Manifold Approximation and Projection (UMAP) plots of high-quality cells (n = 49,064) from all enrolled samples (n = 6) visualizing celltype clusters. (B, B cell; Endo, Endothelial cell; Macrophage, Macrophage cell; NK,Natural Killer cell; Mast cell; T, T cell; VSMCs, Vascular Smooth Muscle cell). **(B)** UMAP plot of atherosclerotic core (AC) and proximal adjacent (PA) distribution. **(C)** Expression levels of marker genes across six major cell types. **(D)** UMAP visualization of expression levels of six key core genes. **(E)** The dot plot illustrates the expression levels of six key core genes across different cell populations in atherosclerotic core and proximal adjacent.

To investigate the expression patterns of six hub genes identified from a dual-disease prognostic model, we analyzed single-cell transcriptomic data comprising 49,064 high-quality cells from six atherosclerotic plaque samples. UMAP clustering revealed six major cell types, including B cells, endothelial cells, macrophages, natural killer (NK) cells, T cells, and vascular smooth muscle cells (VSMCs) (**Figure 7A**). Cells were further stratified by tissue origin into atherosclerotic core (AC) and proximal adjacent (PA) regions (**Figure 7B**). Canonical marker gene expression was used to validate the accuracy of cell type annotation (**Figure 7C**). UMAP plots visualized the distribution of the six core genes across different cell populations (**Figure 7D**). Dot plot analysis further revealed that *TKT* was relatively highly expressed in macrophages, particularly within the atherosclerotic core. In contrast, *KPNA2* and the other four genes exhibited generally low expression levels across most cell types and regions (**Figure 7E**).

### 3.6 Transcription factor analysis and GeneMANIA analysis of six genes

**Figure 8:**
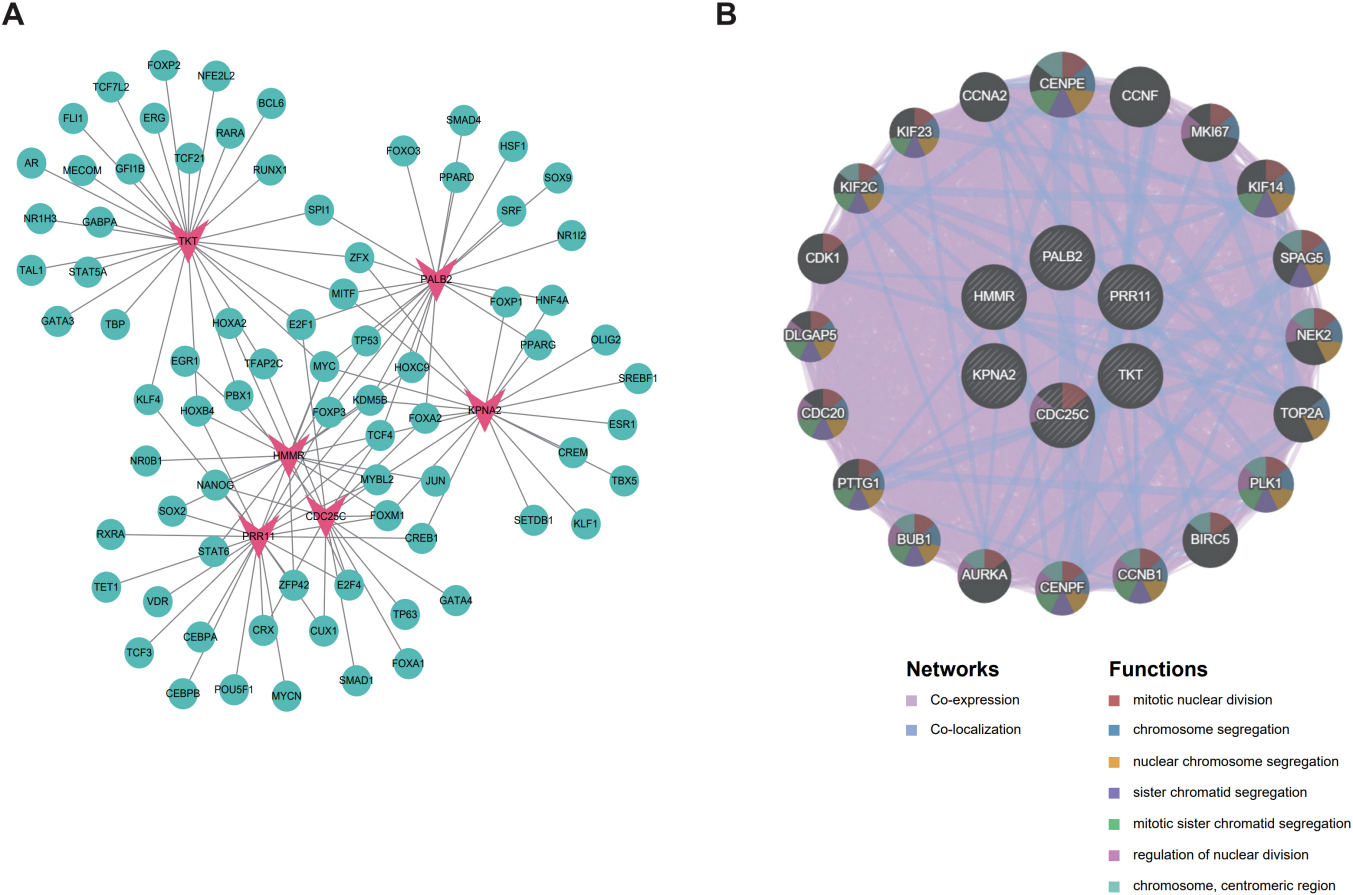
Transcription factors (TFs)-mRNA regulatory network and GeneMANIA analysis of six comorbidity genes shared by CRC and AS. **(A)** TF-mRNA regulatory network, with red indicating biomarkers and green indicating TFs; **(B)** The inner circle represents the six comorbidity biomarkers, the outer circle denotes genes with strong interactions with them, and the inner part of the circle shows the proportions of the top 7 functions.

A total of 76 transcription factors (TFs) were predicted by TF analysis, among which *TKT* corresponded to 27 TFs, *PRR11* to 23, *PALB2* to 21, *KPNA2* to 19, *HMMR* to 17, and *CDC25C* to 15 (**Figure 8A**). GeneMANIA analysis was performed to construct the interaction network of the above six query genes. Results showed that these genes were mainly associated through co-expression networks (accounting for 86.08%) and significantly enriched in biological processes such as mitotic nuclear division and chromosome segregation. The network further predicted 20 highly associated genes (e.g., *CENPE*, *PLK1*), which collectively formed a core gene module involved in cell cycle regulation (**Figure 8B**).

### 3.7 Validation of hub genes expression patterns in CRC and AS by RT-qPCR, Western blot, and IHC staining

**Figure 9:**
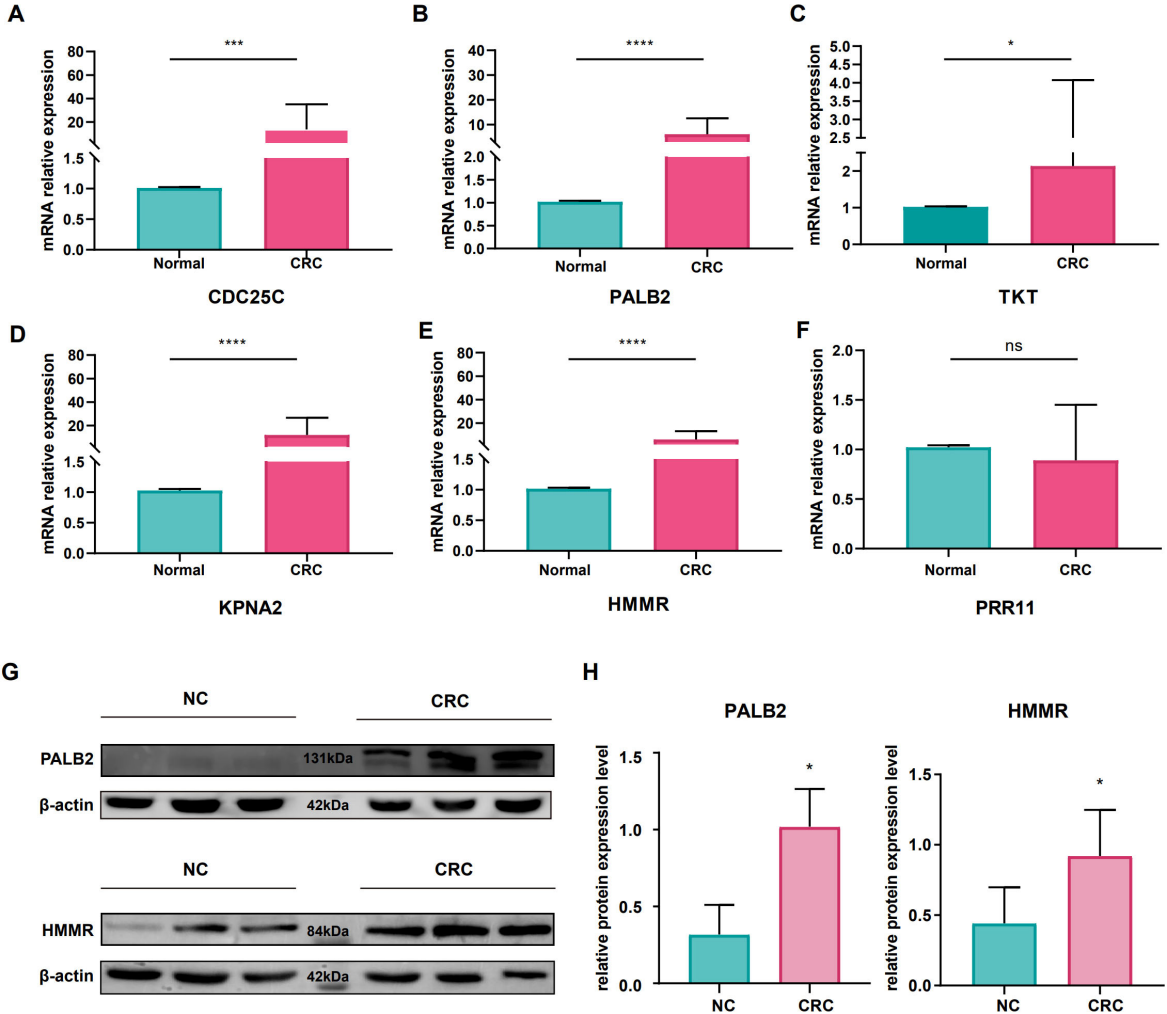
Validation of the expression of 6 hub genes in CRC and AS by molecular biology experiments. **(A-F)** RT-qPCR validation of the differential expression of prognostic genes between normal and tumor tissues in CRC patients. **(G)** Western blot validation of the differential expression of 2 newly identified genes (HMMR and PALB2) between normal and tumor tissues in CRC. **(H)** Quantitative analysis of the relative grayscale values of Western blot bands for the 2 newly identified genes (HMMR and PALB2) in normal and tumor tissues of CRC. Data are presented as mean ± standard deviation (SD), with statistical significance indicated as *p < 0.05, ***p < 0.001, and ****p < 0.0001. ns indicates no statistical significance.

We validated the expression levels of these prognostic genes (*CDC25C*, *HMMR*, *KPNA2*, *PRR11*, *PALB2*, and *TKT*) in CRC patients using qRT-PCR with 23 paired specimens. Comparative analysis revealed that *PRR11* expression did not differ significantly between tumor and normal tissues. In contrast, *HMMR*, *KPNA2*, and *PALB2* were significantly overexpressed in tumor tissues relative to normal counterparts (*p* < 0.0001), while *CDC25C* showed significant overexpression with a slightly lower statistical significance (*p* < 0.001) and *TKT* exhibited a significant overexpression at a moderate level (*p* < 0.05) (**Figures 9A-F**).

Western blot was performed to verify the expression patterns of *HMMR* and *PALB2*—two newly identified potential pathogenic genes for AS—in CRC. A total of 6 CRC patients’ postoperative specimens were included for statistical analysis, and results of 3 representative patients are shown in **Figures 9G-H**. Compared with adjacent normal tissues, both genes were highly expressed in tumor tissues, with statistically significant intergroup differences in gray values (*p* < 0.05; detailed gray value statistical results are presented in **Supplementary Table S3**).

**Figure 10:**
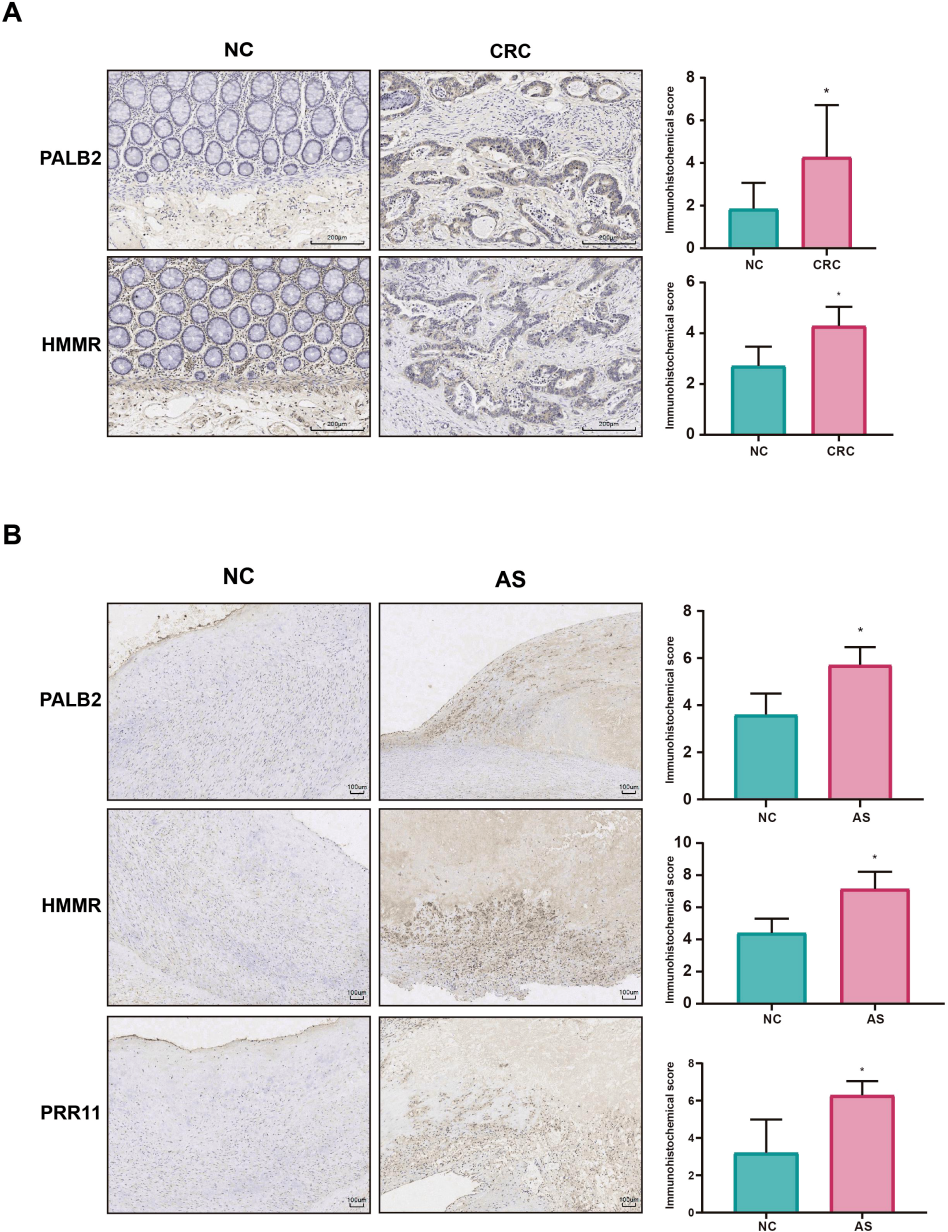
IHC staining validation. **(A)** The differential expression of *PALB2* and *HMMR* in CRC tissues compared with normal tissues; **(B)** displays the differential expression of *HMMR*, *PALB2* and *PRR11* in AS tissues versus normal tissues. *p < 0.05.

Furthermore, IHC staining was performed to evaluate the expression levels of the aforementioned genes: *HMMR* and *PALB2* in CRC tissues, and all three genes in AS tissues. For CRC, 7 tumor tissues and 7 adjacent normal tissues (controls) were included; for AS, 7 lesioned tissues and 3 adjacent non-lesioned tissues (controls) were analyzed, with detailed scores in **Supplementary Table S4**. Results showed *HMMR* and *PALB2* were significantly overexpressed in CRC tumor vs. normal tissues (**Figure 10A**). In AS, all three genes were notably elevated in lesioned vs. non-lesioned tissues (**Figure 10B**), with all group differences statistically significant (*p* < 0.05).

## 4 Discussion

Given the shared phenotypic characteristics and clinical associations between CRC and AS, this study identified 144 potential comorbidity genes for CRC and AS through integrated transcriptome analysis. Based on the survival time and status data of CRC patients, we screened out 6 core comorbidity genes (*CDC25C*, *HMMR*, *KPNA2*, *PRR11*, *PALB2*, and *TKT*) by means of univariate COX regression and LASSO regression, and constructed a CRC risk model. After risk stratification, we performed Gene Set Enrichment Analysis (GSEA) on the high- and low-risk groups, including Kyoto Encyclopedia of Genes and Genomes (KEGG) pathway enrichment analysis and Hallmark gene set enrichment analysis. The diagnostic efficacy and expression levels of these genes in both diseases were systematically evaluated and verified using receiver operating characteristic (ROC) curves and multiple transcriptome datasets. Finally, the expression of these genes in clinical specimens was validated by classical molecular biology experiments, such as quantitative real-time polymerase chain reaction (RT-qPCR), Western blotting (WB), and immunohistochemistry (IHC).

KEGG enrichment analysis of the 144 potential comorbidity genes revealed their core function in “cell cycle regulation”: they affect cell cycle progression by participating in biological processes (e.g., nuclear division, chromosome segregation, spindle function) and molecular events (e.g., DNA/RNA catalysis, kinase activity). It is thus speculated that these genes may mediate the comorbidity association between CRC and AS through the pathway of “cell cycle disorder → abnormal proliferation of tumor cells (driving CRC progression) and imbalance of vascular cell repair/proliferation (promoting AS progression)”. In addition, the enrichment results of cell cycle and DNA replication pathways suggest that abnormal cell proliferation may be a key common driver of both diseases. Future studies can focus on biological processes (BP) such as cell cycle pathways, nuclear division/chromosome segregation, and molecular functions (MF) such as tubulin binding/kinase activity to further verify gene functions and disease mechanisms.

Notably, in the assessment of CRC-related death risk, we identified 24 genes that act as protective factors, including the 6 core genes screened out later. This consistent protective property indicates that they may share common biological mechanisms in inhibiting CRC progression. As key molecules in CRC, their core functions are concentrated in cell cycle regulation, involving critical biological processes such as nuclear division, chromosome segregation, and spindle function, as well as molecular events including DNA/RNA catalysis and kinase activity. This provides a clear biological basis for their role as protective factors—participating in maintaining the normal progression of the cell cycle may be the core mechanism by which they reduce CRC-related death risk.

Among the 24 protective genes, the aforementioned 6 core comorbidity genes were screened out using two machine learning algorithms: univariate COX regression and LASSO regression. These genes inherit the protective properties of this gene set, and higher expression levels are associated with better prognosis in CRC patients. The risk model constructed based on these 6 genes showed reliable predictive efficacy in both the training set and validation set (area under the curve (AUC) = 0.63-0.67 in the training set; AUC = 0.57-0.75 in the validation set). Notably, the predictive efficacy of this model is comparable to that of other CRC prognostic models constructed based on the cell cycle (20, 21), which not only confirms the scientificity and reliability of constructing CRC prognostic models based on cell cycle regulation-related genes but also reflects the reference value of the model in this study for CRC risk assessment Meanwhile, this model not only verifies the prognostic value of the 6 core genes but also highlights the potential clinical significance of the 24 protective genes in CRC risk assessment and management. The CRC risk model constructed based on these genes has reliable prognostic efficacy, and the differences in immune checkpoints revealed by risk stratification provide potential targets for personalized treatment of CRC, as well as new clues for the study of AS pathogenesis and the screening of therapeutic targets.

GSEA-KEGG pathway and Hallmark enrichment analyses of the high- and low-risk groups showed that the pathway characteristics of the low-risk group may reflect the body’s ability to maintain tissue homeostasis through metabolic regulation, matrix remodeling, and angiogenesis, which may be an important reason for their favorable prognosis. In contrast, the enrichment of abnormal DNA repair pathways and immune dysregulation in the high-risk group may be associated with increased genomic instability, enhanced drug resistance, and immune escape of tumor cells, thereby promoting tumor progression and providing a biological basis for explaining the poor prognosis of the high-risk group. These differential pathways can serve as potential targets for CRC risk stratification and targeted therapy.

Immune cell infiltration assessment based on CIBERSORT showed significant differences in the proportion of immune cells between the high- and low-risk groups: the low-risk group was enriched in follicular helper T cells and activated dendritic cells, which can enhance anti-tumor immune responses; the high-risk group was dominated by memory B cells and resting mast cells, with relatively insufficient infiltration of immune effector cells. Meanwhile, immune checkpoint analysis showed that *CD276*, *ADORA2A*, *TNFRSF4*, and *TNFRSF14* were significantly upregulated in the high-risk group (*p* < 0.05), indicating a more pronounced immunosuppressive microenvironment. These findings not only explain the prognostic differences between the two groups from an immunological perspective but also suggest that these immune cell characteristics and immune checkpoint molecules can serve as potential targets for CRC risk stratification and targeted immunotherapy.

The results of drug sensitivity analysis between risk groups further confirmed the core hypothesis that “risk stratification is closely associated with cell cycle regulation”: the four drugs with significant sensitivity differences between high- and low-risk groups all target key links of the cell cycle. Specifically, BI-2536 targets *PLK1*, a core kinase of mitosis (G2/M phase) (22); MK-1775 acts on *Wee1*, a key molecule of the G2/M phase DNA damage checkpoint (23); Dihydrorotenone can induce G0/G1 phase cell cycle arrest (24); and TAF1, as an upstream transcriptional regulator, modulates the expression of cell cycle-related genes such as *cyclin* and *CDK (*25). These drugs cover the entire cell cycle from initiation to division, directly confirming that the selected drugs are all associated with cell cycle regulation.

Multi-omics-based bioinformatics analysis demonstrated that these six key comorbidity genes exhibit reliable predictive efficacy in both CRC and AS, while also uncovering their expression patterns in the two diseases. Notably, the observed expression differences of *PRR11* across different datasets and experimental validations are considered to be mainly associated with the following four aspects of factors: firstly, regarding the TCGA-COADREAD dataset, its samples are mainly derived from the University of North Carolina, USA, and exon expression profiles are detected based on the Illumina HiSeq 2000 RNA sequencing platform, which relies on high-throughput next-generation sequencing (NGS) technology to directly capture the raw sequence information of *PRR11* transcripts (26); secondly, the GSE39582 dataset consists of samples from the CIT program cohort of the French Ligue Nationale Contre le Cancer, and adopts the Affymetrix GPL570 gene chip technology, with its detection process relying on the hybridization reaction between pre-designed oligonucleotide probes and target RNA (27); taken together, the differences in the technical principles of the detection platforms and the population heterogeneity in sample origins (covering the USA and France) synergistically contribute to the observed expression differences of *PRR11* across different dataset.

Previous studies have demonstrated that *CDC25C*, *HMMR*, *KPNA2*, and *PRR11* directly participate in the regulation of cell cycle progression (28–30); *PALB2* indirectly affects cell cycle regulation through homologous recombination repair (31); and *TKT* is a key gene in the pentose phosphate pathway (PPP) (32). These studies confirm that the key pathways regulated by these genes are closely associated with lipid metabolic reprogramming and immune microenvironment remodeling, which supports the hypothesis of phenotypic commonalities between the two diseases proposed in the introduction and provides a basis for exploring their comorbidity mechanism.

Based on the comprehensive analysis of these six comorbidity-related genes, *KPNA2* and *TKT* deserve special attention due to their higher expression levels at the single-cell level. Notably, *TKT* exhibits high expression abundance in both CRC epithelial cells and AS macrophages, and this cell-specific expression profile provides a more precise localization for its role in the two diseases: In CRC, epithelial cells are the core site of tumorigenesis, and high *TKT* expression may enhance the activity of the PPP to supply sufficient nucleotides and nicotinamide adenine dinucleotide phosphate (*NADPH*) for the rapid proliferation of tumor epithelial cells—with the former being a key raw material for DNA synthesis and the latter involved in maintaining intracellular redox balance and lipid synthesis, thereby meeting the metabolic demands of tumor cells (33); In AS, macrophages are key immune cells mediating chronic inflammation and vascular injury, and high *TKT* expression in macrophages may influence the polarization phenotype of macrophages by regulating the PPP pathway, such as promoting the activation of M1 pro-inflammatory macrophages, enhancing the secretion of pro-inflammatory factors (e.g., tumor necrosis factor-α (*TNF-α*), interleukin-6 (*IL-6*), and participating in foam cell formation by affecting the production of lipid metabolic intermediates (34), thereby promoting vascular wall inflammation and atherosclerotic progression in AS. This cell-specific high-expression pattern suggests that *TKT* may act as a key molecule linking the abnormal proliferation of CRC epithelial cells and the macrophage-mediated inflammatory response in AS by regulating tumor cell metabolism and immune cell function respectively, further supporting the core role of the “cell cycle-lipid metabolism-immunity” crosstalk regulatory network in the comorbidity mechanism of the two diseases.

As a nuclear transport protein, *KPNA2* regulates oxidative stress and ferroptosis pathways by mediating the nuclear translocation of nuclear factor erythroid 2-related factor 2 (*NRF2*) in non-small cell lung cancer (35), and simultaneously participates in lipid metabolic reprogramming to provide energy and material basis for tumor cell proliferation; in addition, it can also involve in the remodeling of the tumor immune microenvironment by promoting the nuclear translocation of key molecules in the nuclear factor-κB (NF-κB) pathway (36). In AS, *KPNA2* drives immune remodeling by mediating the nuclear translocation of p65 and interferon regulatory factor 3 (*IRF3*), thereby enhancing the secretion of pro-inflammatory factors by vascular endothelial cells and monocyte adhesion (37).

*CDC25C* is a core regulatory factor for G2/M phase transition. It drives cells into mitosis by activating the cyclin-dependent kinase 1 (*CDK1*)/*cyclin B1* complex, and further participates in lipid metabolic reprogramming and immune microenvironment remodeling (38). In lipid metabolism, its high expression indirectly supports lipid synthesis and metabolism by maintaining cell cycle progression; for instance, in pancreatic adenocarcinoma, inhibition of *CDC25C* impairs mitochondrial respiratory function and increases reactive oxygen species (*ROS*) accumulation (39), suggesting that it may affect the energy supply for lipid metabolism by regulating mitochondrial homeostasis. In addition, *CDC25C* is upregulated in lung adenocarcinoma (LUAD), and its high expression is significantly associated with immune cell infiltration and immune-related characteristics in the tumor microenvironment, as well as shortened progression-free survival in LUAD patients receiving nivolumab treatment, indicating a correlation between *CDC25C* and immunotherapeutic efficacy as well as the tumor immune microenvironment (40). *CDC25C* is a core molecule regulating the G2/M phase transition of vascular smooth muscle cell (VSMC) cycle in AS, which induces cell cycle arrest through the ATM-CHK2-Cdc25C-p21WAF1-Cdc2 cascade and participates in bisphenol A (*BPA*)-induced cardiovascular damage (41).

*HMMR* and *PALB2*, two novel potential AS-causative genes identified in this study, also play key roles in CRC progression, offering new insights into the comorbidity mechanism of these two diseases. *HMMR*, a hyaluronan-mediated motility receptor, primarily regulates cell migration and proliferation via hyaluronan binding. In CRC, it activates PI3K/Akt, MAPK, and other pathways to promote epithelial-mesenchymal transition (EMT) and tumor angiogenesis (42). *HMMR* also associates with immune infiltration in lung adenocarcinoma (43), implying a role in CRC progression via immune microenvironment regulation. No reports link *HMMR* to lipid metabolic reprogramming; its potential AS role may focus on vascular cell proliferation and immune-inflammatory responses.

*PALB2*, a key protein in homologous recombination repair, maintains genomic stability in CRC through *BRCA2* interaction. Abnormal *PALB2* expression or mutation increases CRC cell sensitivity to DNA-damaging chemotherapeutics (e.g., oxaliplatin) and correlates with CRC risk and prognosis (44). Additionally, *PALB2* mediates hepatocellular carcinoma immune microenvironment remodeling (dual regulation of immune suppression and T-cell infiltration) via the cGAS-STING pathway, depending on its binding status with *BRCA1 (*45)—a mechanism that may also regulate the CRC immune microenvironment. No evidence supports direct or indirect lipid metabolism regulation by *PALB2*; as a potential AS-causative gene, it may contribute to AS progression via crosstalk between genomic stability maintenance and immune-inflammatory regulation.

*PRR11*, another novel potential AS-causative gene identified herein, exhibits complex functions and expression patterns in CRC. As a key promoter of G2/M phase progression, high *PRR11* expression in CRC accelerates cell cycle and promotes abnormal proliferation by regulating *Cyclin B1/CDK1* activity (46). In glioblastoma, *PRR11* inhibits ferroptosis by stabilizing dihydroorotate dehydrogenase (*DHODH*) (47), suggesting indirect links to lipid peroxidation in lipid metabolism. *PRR11* also associates with immune infiltration in bladder cancer (48) a potential immune-regulatory role that may extend to CRC, though its mechanism remains unclear.

Transcription factor (TF) analysis predicted 76 regulatory TFs, with the 6 target genes forming a complex transcriptional network via shared TFs. Gene pairs including *HMMR*-*KPNA2* and *KPNA2*-*PALB2* shared 5 and 8 TFs, respectively. Core TFs (MYC, TCF4, FOXM1, E2F family) cross-regulate multiple target genes, acting as key upstream factors maintaining network coordination. GeneMANIA analysis showed the 6-gene interaction network is dominated by co-expression relationships (86.08%) and enriched in core cell cycle processes (e.g., mitotic nuclear division, chromosome segregation). The network’s 20 highly associated genes (e.g., *CENPE*, *PLK1*) form a cell cycle regulatory module with the 6 targets, jointly regulating cell cycle progression.

Given the mRNA-based sequencing data, we initially employed RT-qPCR to measure 6-gene expression in 20 pairs of CRC matched specimens from Sun Yat-sen Memorial Hospital (Guangdong, China). Using specific primers targeting *PRR11’s* core coding region for precise transcript quantification, we found 5 genes (except *PRR11*) were significantly upregulated in CRC tissues versus adjacent normal tissues. *PRR11* showed no significant difference and inconsistent expression across datasets, attributed to population heterogeneity (samples from the US, France, Guangdong, China), limited sample size (20 pairs insufficient for accurate characterization), detection platform differences (NGS, gene chip, RT-qPCR), and weak correlation between its CRC protein level and prognosis. Larger cohorts are needed to clarify its pathogenic mechanism.

Since *CDC25C*, *TKT*, and *KPNA2* have established roles in CRC and AS, we initially selected *HMMR*, *PALB2*, and *PRR11* (unreported in AS) for cross-disease validation. *PRR11* was excluded from subsequent WB and IHC assays due to its expression heterogeneity and poor prognostic relevance, ensuring research clarity. Due to limited AS tissue availability, only IHC was used for AS protein validation, while CRC tissues underwent dual WB/IHC verification. Results showed the 3 genes were upregulated in AS lesions (consistent with multi-omics trends), suggesting pro-AS effects; *HMMR* and *PALB2* also showed high expression in CRC, supporting their potential as CRC prognostic markers.

Despite shared pathological phenotypes and causative genes, no causal relationship between CRC and AS has been confirmed—existing evidence supports a parallel association. In summary, we identified 5 core comorbidity genes for CRC and AS, and hypothesize they mediate both diseases via the “cell cycle-lipid metabolism-immunity” crosstalk network, providing new insights into “tumor-vascular disease” comorbidity. This hypothesis, however, remains unvalidated by molecular or animal experiments.

This study has limitations: 1) The signature genes used to classify CRC high-/low-risk groups overlap with the core gene sets for subsequent GSEA and immune infiltration analyses, which may lead to a superposition effect of correlation signals; 2) The study relies on retrospective databases such as TCGA (predominantly including European and American populations), and the sample size related to AS is relatively limited. Notably, validation in independent Asian cohorts is lacking, which may limit the generalizability of the conclusions across different populations; 3) Functional analysis is centered on bioinformatics predictions, lacking functional verification through cellular and animal experiments; the risk model does not incorporate clinicopathological features such as tumor stage, leaving room for improvement in the accuracy of risk stratification. Future research could use multi-center prospective cohorts, genetically engineered animal models, and molecular assays to verify the 6 key genes’ expression and roles in the “cell cycle-lipid metabolism-immunity” regulatory network. Additional studies could explore causality by inducing CRC in AS models (or vice versa) or using cell cycle-deficient models to test lipid metabolic and immune phenotypes, while integrating clinical follow-up investigations to further validate the proposed mechanism and clarify the deep-seated association between the two diseases.

## 5 Conclusion

Firstly, *CDC25C*, *HMMR*, *KPNA2*, *PALB2*, and *TKT* are common pathogenic genes for CRC and AS, and *HMMR*, *PALB2* and *PRR11* are expected to be potential new targets for AS.

Secondly, after constructing a CRC risk model based on these 6 genes and performing grouping, GSEA analysis showed that the KEGG and Hallmark pathways enriched in the high-risk group are associated with immune microenvironment remodeling and metabolic reprogramming. Immune infiltration analysis revealed that with increasing risk, the low-risk group had more abundant follicular helper T (Tfh) cells and activated dendritic cells, while the high-risk group was dominated by memory B cells and resting mast cells. Immune checkpoint analysis suggested that *CD276*, *ADORA2A*, *TNFRSF4*, and *TNFRSF14* may help delay disease progression in high-risk CRC patients. High- and low-risk groups exhibited significant IC_50_ differences for BI-2536, MK-1775, Dihydrorotenone and TAF1-targeting agents in drug sensitivity analysis.

Thirdly, validation via spatial transcriptomics, multi-omics, and molecular biology experiments confirmed that the expression levels of these 6 genes in CRC and AS lesional tissues were generally significantly higher than those in adjacent non-lesional tissues, except for *PRR11*, which showed lower expression in CRC tumor tissues in dataset GSE39582 and RT-qPCR validation.

Finally, the CRC risk model constructed in this study and the identified cross-disease targets are expected to provide new ideas for the clinical diagnosis and treatment of CRC and AS.

## Data Availability

The datasets generated and analyzed during the current study are available in the TCGA portal (https://portal.gdc.cancer.gov/) with dataset ID TCGA-COADREAD, and in the GEO repository (https://www.ncbi.nlm.nih.gov/geo/) with dataset IDs: GSE87211, GSE10950, GSE39582, GSE132465, GSE225857, GSE100927, GSE43292, and GSE159677. For details, please refer to **Supplementary Table S1** in the Supplementary Materials.

## References

[1] Sullivan BA Noujaim M Roper J . Cause, epidemiology, and histology of polyps and pathways to colorectal cancer. Gastrointest Endosc Clin N Am. (2022) 32:177–94. doi: 10.1016/j.giec.2021.12.001, PMID: 35361330 PMC9924026

[2] Goto A Yamaji T Sawada N Momozawa Y Kamatani Y Kubo M . Diabetes and cancer risk: A Mendelian randomization study. Int J Can. (2020) 146:712–9. doi: 10.1002/ijc.32310, PMID: 30927373 PMC6916579

[3] Du W Xia X Gou Q Qiu Y . Mendelian randomization and transcriptomic analysis reveal a positive cause-and-effect relationship between Alzheimer’s disease and colorectal cancer. Transl Oncol. (2025) 51:102169. doi: 10.1016/j.tranon.2024.102169, PMID: 39608211 PMC11635780

[4] Chen H Zheng X Zong X Li Z Li N Hur J . Metabolic syndrome, metabolic comorbid conditions and risk of early-onset colorectal cancer. Gut. (2021) 70:1147–54. doi: 10.1136/gutjnl-2020-321661, PMID: 33037055 PMC8032822

[5] Libby P . The changing landscape of atherosclerosis. Nature. (2021) 592:524–33. doi: 10.1038/s41586-021-03392-8, PMID: 33883728

[6] Rzasa P Rufini A . Cholesterol metabolism and colorectal cancer: the plot thickens. Cell Death Discov. (2024) 10:3. doi: 10.1038/s41420-023-01784-5, PMID: 38177141 PMC10766946

[7] Xiong L Liu HS Zhou C Yang X Huang L Jie HQ . A novel protein encoded by circINSIG1 reprograms cholesterol metabolism by promoting the ubiquitin-dependent degradation of INSIG1 in colorectal cancer. Mol Can. (2023) 22:72. doi: 10.1186/s12943-023-01773-3, PMID: 37087475 PMC10122405

[8] Seeley EH Liu Z Yuan S Stroope C Cockerham E Rashdan NA . Spatially resolved metabolites in stable and unstable human atherosclerotic plaques identified by mass spectrometry imaging. Arterioscler Thromb Vasc Biol. (2023) 43:1626–35. doi: 10.1161/ATVBAHA.122.318684, PMID: 37381983 PMC10527524

[9] Berndsen ZT Cassidy CK . The structure of apolipoprotein B100 from human low-density lipoprotein. Nature. (8051) 2025:638. doi: 10.1038/s41586-024-08467-w, PMID: 39662503 PMC11839476

[10] Pi H Wang G Wang Y Zhang M He Q Zheng X . Immunological perspectives on atherosclerotic plaque formation and progression. Front Immunol. (2024) 15:1437821. doi: 10.3389/fimmu.2024.1437821, PMID: 39399488 PMC11466832

[11] Overacre-Delgoffe AE Bumgarner HJ Cillo AR Burr AHP Tometich JT Bhattacharjee A . Microbiota-specific T follicular helper cells drive tertiary lymphoid structures and anti-tumor immunity against colorectal cancer. Immunity. (2021) 54:2812–24 e4. doi: 10.1016/j.immuni.2021.11.003, PMID: 34861182 PMC8865366

[12] Mizuno R Kawada K Itatani Y Ogawa R Kiyasu Y Sakai Y . The role of tumor-associated neutrophils in colorectal cancer. Int J Mol Sci. 529. doi: 10.3390/ijms20030529, PMID: 30691207 PMC6386937

[13] Qiu H Wang L Zhou L Wang X . Comorbidity patterns in patients newly diagnosed with colorectal cancer: network-based study. JMIR Public Health Surveill. (2023) 9:e41999. doi: 10.2196/41999, PMID: 37669093 PMC10509734

[14] Levy JA Kazemian E Ramin C Lorona NC Nadri M Gasho JO . Subclinical atherosclerosis and cardiovascular events among patients with colorectal cancer. Cancer Med. (2025) 14:e70938. doi: 10.1002/cam4.70938, PMID: 40365909 PMC12076194

[15] Dzaye O Berning P Dardari ZA Mortensen MB Marshall CH Nasir K . Coronary artery calcium is associated with increased risk for lung and colorectal cancer in men and women: the Multi-Ethnic Study of Atherosclerosis (MESA). Eur Heart J Cardiovasc Imag. (2022) 23:708–16. doi: 10.1093/ehjci/jeab099, PMID: 34086883 PMC9016360

[16] Nehme F . An intimal relationship between atherosclerosis and colorectal cancer screening. Dig Dis Sci. (2020) 65:1588–9. doi: 10.1007/s10620-020-06046-3, PMID: 31907770

[17] Tang D Chen M Huang X Zhang G Zeng L Zhang G . SRplot: A free online platform for data visualization and graphing. PloS One. (2023) 18:e0294236. doi: 10.1371/journal.pone.0294236, PMID: 37943830 PMC10635526

[18] Shen W Song Z Zhong X Huang M Shen D Gao P . Sangerbox: A comprehensive, interaction-friendly clinical bioinformatics analysis platform. Imeta. (2022) 1:e36. doi: 10.1002/imt2.36, PMID: 38868713 PMC10989974

[19] Zhang X Zhang X Li G Hao Y Liu L Zhang L . A novel necroptosis-associated lncRNA signature can impact the immune status and predict the outcome of breast cancer. J Immunol Res. (2022) 2022:3143511. doi: 10.1155/2022/3143511, PMID: 35578667 PMC9107037

[20] Gu C Jin L Lv X Wang C Wen C Su X . Development and validation of a prognostic model for colon cancer based on mitotic gene signatures and immune microenvironment analysis. Discov Oncol. (2024) 15:535. doi: 10.1007/s12672-024-01421-2, PMID: 39382813 PMC11464972

[21] Hou Y Zhang R Zong J Wang W Zhou M Yan Z . Comprehensive analysis of a cancer-immunity cycle-based signature for predicting prognosis and immunotherapy response in patients with colorectal cancer. Front Immunol. (2022) 13:892512. doi: 10.3389/fimmu.2022.892512, PMID: 35711437 PMC9193226

[22] Gohda J Suzuki K Liu K Xie X Takeuchi H Inoue JI . BI-2536 and BI-6727, dual Polo-like kinase/bromodomain inhibitors, effectively reactivate latent HIV-1. Sci Rep. (2018) 8:3521. doi: 10.1038/s41598-018-21942-5, PMID: 29476067 PMC5824842

[23] Zhang C Peng K Liu Q Huang Q Liu T . Adavosertib and beyond: Biomarkers, drug combination and toxicity of WEE1 inhibitors. Crit Rev Oncol Hematol. (2024) 193:104233. doi: 10.1016/j.critrevonc.2023.104233, PMID: 38103761

[24] Xu X Zhang J Han K Zhang Z Chen G Zhang J . Natural pesticide dihydrorotenone arrests human plasma cancer cells at the G0/G1 phase of the cell cycle. J Biochem Mol Toxicol. (2014) 28:232–8. doi: 10.1002/jbt.21558, PMID: 24615755

[25] Crombie EM Cleverley K Timmers HTM Fisher EMC . The roles of TAF1 in neuroscience and beyond. R Soc Open Sci. (2024) 11:240790. doi: 10.1098/rsos.240790, PMID: 39323550 PMC11423858

[26] Cancer Genome Atlas N . Comprehensive molecular characterization of human colon and rectal cancer. Nature. (2012) 487:330–7. doi: 10.1038/nature11252, PMID: 22810696 PMC3401966

[27] Marisa L de Reynies A Duval A Selves J Gaub MP Vescovo L . Gene expression classification of colon cancer into molecular subtypes: characterization, validation, and prognostic value. PloS Med. (2013) 10:e1001453. doi: 10.1371/journal.pmed.1001453, PMID: 23700391 PMC3660251

[28] Gousias K Theocharous T Simon M . Mechanisms of cell cycle arrest and apoptosis in glioblastoma. Biomedicines. (2022) 10. doi: 10.3390/biomedicines10030564, PMID: 35327366 PMC8945784

[29] Liu K Zheng M Lu R Du J Zhao Q Li Z . The role of CDC25C in cell cycle regulation and clinical cancer therapy: a systematic review. Cancer Cell Int. (2020) 20:213. doi: 10.1186/s12935-020-01304-w, PMID: 32518522 PMC7268735

[30] Ji Y Xie M Lan H Zhang Y Long Y Weng H . PRR11 is a novel gene implicated in cell cycle progression and lung cancer. Int J Biochem Cell Biol. (2013) 45:645–56. doi: 10.1016/j.biocel.2012.12.002, PMID: 23246489

[31] Jiang M Jia K Wang L Li W Chen B Liu Y . Alterations of DNA damage response pathway: Biomarker and therapeutic strategy for cancer immunotherapy. Acta Pharm Sin B. (2021) 11:2983–94. doi: 10.1016/j.apsb.2021.01.003, PMID: 34729299 PMC8546664

[32] Tong L Chen Z Li Y Wang X Yang C Li Y . Transketolase promotes MAFLD by limiting inosine-induced mitochondrial activity. Cell Metab. (2024) 36:1013–29 e5. doi: 10.1016/j.cmet.2024.03.003, PMID: 38547864

[33] Giacomini I Ragazzi E Pasut G Montopoli M . The pentose phosphate pathway and its involvement in cisplatin resistance. Int J Mol Sci. (2020) 21. doi: 10.3390/ijms21030937, PMID: 32023830 PMC7036764

[34] Malekmohammad K Bezsonov EE Rafieian-Kopaei M . Role of lipid accumulation and inflammation in atherosclerosis: focus on molecular and cellular mechanisms. Front Cardiovasc Med. (2021) 8:707529. doi: 10.3389/fcvm.2021.707529, PMID: 34552965 PMC8450356

[35] Zhang L Xu Y Cheng Z Zhao J Wang M Sun Y . The EGR1/miR-139/NRF2 axis orchestrates radiosensitivity of non-small-cell lung cancer via ferroptosis. Cancer Lett. (2024) 595:217000. doi: 10.1016/j.canlet.2024.217000, PMID: 38821254

[36] Peng N Yang X Zhu C Zhou L Yu H Li M . MicroRNA-302 cluster downregulates enterovirus 71-induced innate immune response by targeting KPNA2. J Immunol. (2018) 201:145–56. doi: 10.4049/jimmunol.1701692, PMID: 29777028

[37] Xing Z Zhen Y Chen J Du M Li D Liu R . KPNA2 silencing, regulated by E3 ubiquitin ligase FBXW7, alleviates endothelial dysfunction and inflammation through inhibiting the nuclear translocation of p65 and IRF3: A possible therapeutic approach for atherosclerosis. Inflammation. (2023) 46:2071–88. doi: 10.1007/s10753-023-01863-w, PMID: 37432596

[38] Xue T Chen Y Xu J Du W Kong P Zhang X . Cyclovirobuxine D inhibits growth and progression of non−small cell lung cancer cells by suppressing the KIF11−CDC25C−CDK1−CyclinB1 G(2)/M phase transition regulatory network and the NFkappaB/JNK signaling pathway. Int J Oncol. (2023) 62:57. doi: 10.3892/ijo.2023.5505, PMID: 36929198 PMC10124714

[39] Zhou C Wang L Hu W Tang L Zhang P Gao Y . CDC25C is a prognostic biomarker and correlated with mitochondrial homeostasis in pancreatic adenocarcinoma. Bioengineered. (2022) 13:13089–107. doi: 10.1080/21655979.2022.2078940, PMID: 35615982 PMC9275923

[40] Zhang W Shang X Yang F Han W Xia H Liu N . CDC25C as a predictive biomarker for immune checkpoint inhibitors in patients with lung adenocarcinoma. Front Oncol. (2022) 12:867788. doi: 10.3389/fonc.2022.867788, PMID: 35574406 PMC9104567

[41] Kim H Park H Hwang B Kim S Choi YH Kim WJ . Bisphenol A exposure inhibits vascular smooth muscle cell responses: Involvement of proliferation, migration, and invasion. Environ Toxicol Pharmacol. (2023) 98:104060. doi: 10.1016/j.etap.2023.104060, PMID: 36610522

[42] Zhang H Ren L Ding Y Li F Chen X Ouyang Y . Hyaluronan-mediated motility receptor confers resistance to chemotherapy via TGFbeta/Smad2-induced epithelial-mesenchymal transition in gastric cancer. FASEB J. (2019) 33:6365–77. doi: 10.1096/fj.201802186R, PMID: 30802150

[43] Ma X Xie M Xue Z Yao J Wang Y Xue X . HMMR associates with immune infiltrates and acts as a prognostic biomaker in lung adenocarcinoma. Comput Biol Med. (2022) 151:106213. doi: 10.1016/j.compbiomed.2022.106213, PMID: 36306573

[44] Yurgelun MB Kulke MH Fuchs CS Allen BA Uno H Hornick JL . Cancer susceptibility gene mutations in individuals with colorectal cancer. J Clin Oncol. (2017) 35:1086–95. doi: 10.1200/JCO.2016.71.0012, PMID: 28135145 PMC5455355

[45] Ma H Kang Z Foo TK Shen Z Xia B . Disrupted BRCA1-PALB2 interaction induces tumor immunosuppression and T-lymphocyte infiltration in HCC through cGAS-STING pathway. Hepatology. (2023) 77:33–47. doi: 10.1002/hep.32335, PMID: 35006619 PMC9271123

[46] Ma H Yang W Wang X Dai G . PRR11 Promotes Proliferation and Migration of Colorectal Cancer through Activating the EGFR/ERK/AKT Pathway via Increasing CTHRC1. Ann Clin Lab Sci. (2022) 52:86–94., PMID: 35181621

[47] Miao Z Xu L Gu W Ren Y Li R Zhang S . A targetable PRR11-DHODH axis drives ferroptosis- and temozolomide-resistance in glioblastoma. Redox Biol. (2024) 73:103220. doi: 10.1016/j.redox.2024.103220, PMID: 38838551 PMC11179629

[48] Ni W Yi L Dong X Cao M Zheng J Wei Q . PRR11 is a prognostic biomarker and correlates with immune infiltrates in bladder urothelial carcinoma. Sci Rep. (2023) 13:2051. doi: 10.1038/s41598-023-29316-2, PMID: 36739300 PMC9899238

