# Supplementary material for: Prognostic value and experimental validation of atherosclerosis-derived pathogenic genes in colorectal cancer
Source: Front Oncol. 2026 Jan 12;15:1728087. doi: 10.3389/fonc.2025.1728087 (PMC12832349; doi:10.3389/fonc.2025.1728087)

The target protein detected in the experiments is

PALB2 with a molecular weight of 131 kDa, and the

internal reference protein is β-actin (42 kDa).

The protein marker used in the experiments was

purchased from Yazyme (Catalog No.: WJ103).

This membrane contains cancer tissues and adjacent normal tissues from 3 colorectal

cancer patients. The developed target band is PALB2 with a molecular weight of 131 kDa,

among which the part framed in red corresponds to the area presented in the first panel of

Figure 8G. The specific lane distribution is as follows: Lanes 1 and 10 contain the

aforementioned Marker; Lanes 5 and 9 contain loading buffer (no sample); Lanes 2-4

represent the paracancerous normal tissues from Patient 1, Patient 2, and Patient 3

respectively; Lanes 6-8 represent the corresponding colorectal cancer tumor tissues from

these three patients.

Maker

Maker

150 kDa

100 kDa

150 kDa

100 kDa

70 kDa

70 kDa

50 kDa

40 kDa

50 kDa

40 kDa

35 kDa

35 kDa

Normal

CRC

PALB2 (131 kDa)


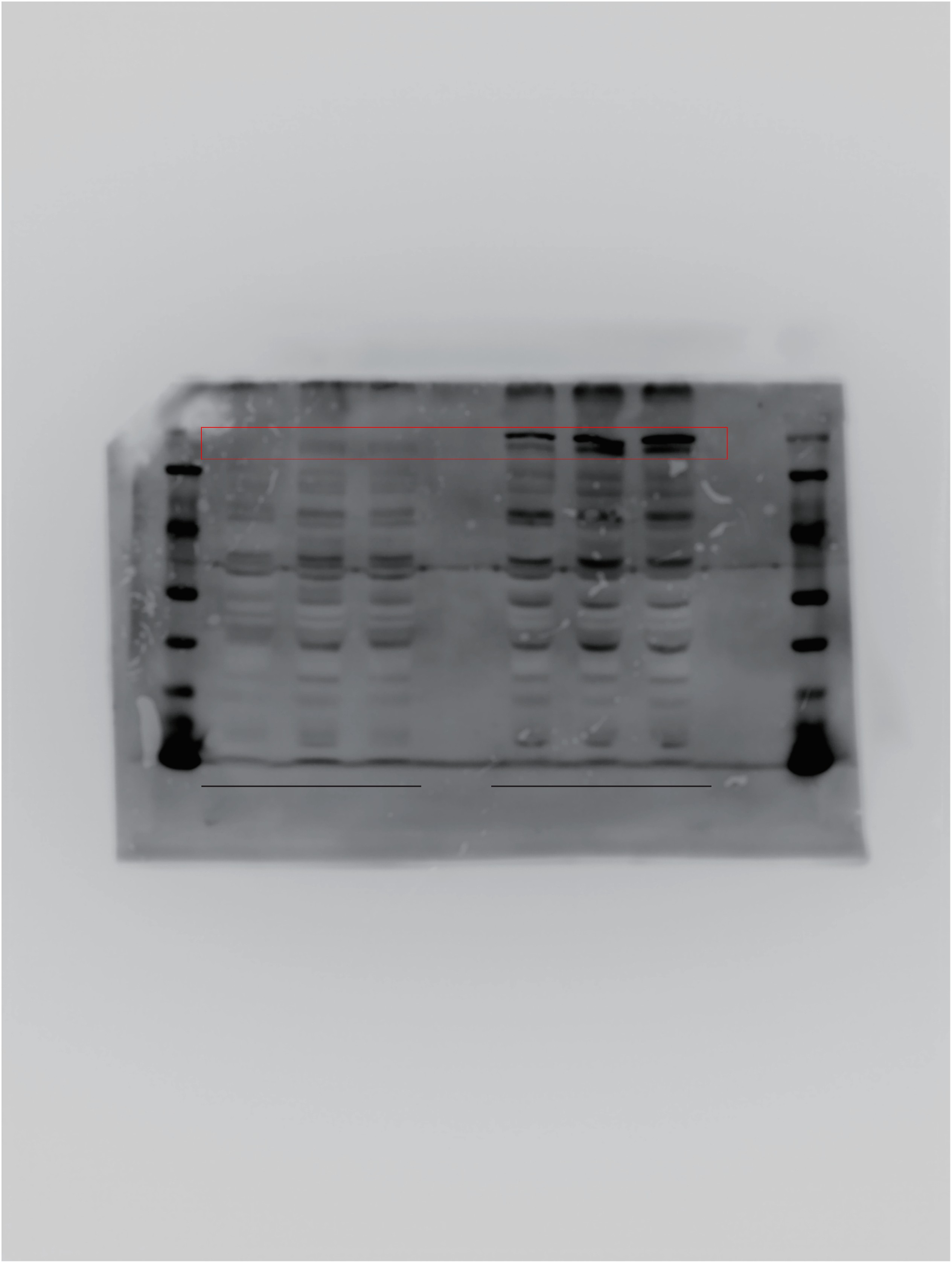


This membrane is the internal reference part of the previous

membrane, obtained by stripping and redeveloping, with β-actin

as the internal reference (molecular weight: 42 kDa); the part

framed in red corresponds to the second panel of Figure 8G. The

Marker, groupings, and lane arrangements are consistent with

those on the previous page.

Maker

Maker

150 kDa

100 kDa

70 kDa

150 kDa

100 kDa

70 kDa

50 kDa

50 kDa

40 kDa

40 kDa

35 kDa

35 kDa

Normal

CRC

β-actin(42 kDa)


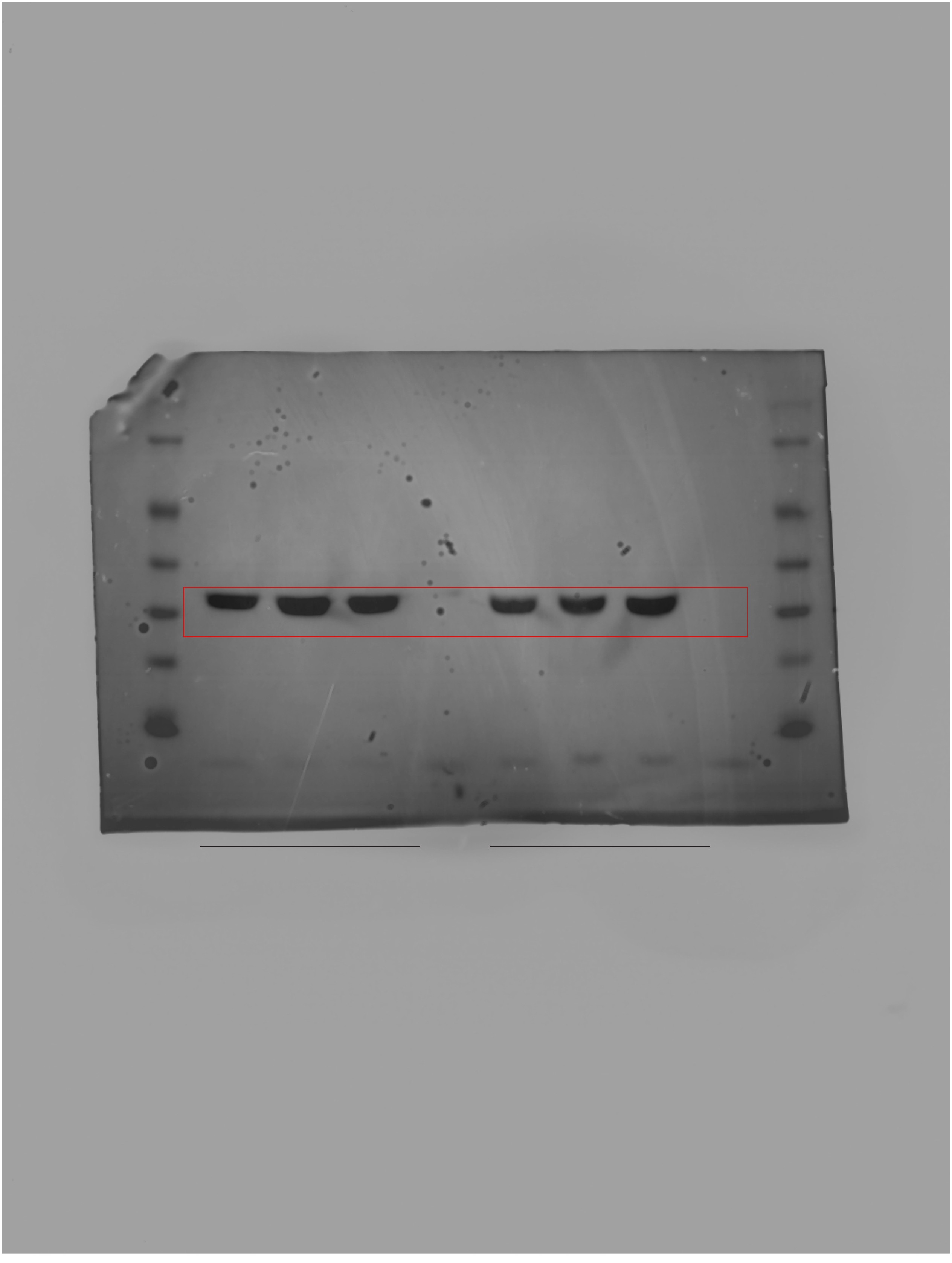


This protein membrane contains the cancerous and paracancerous normal tissues from

colorectal cancer patients No. 4-6. The target band detected in this experiment is PALB2

(molecular weight: 131 kDa), with the area enclosed in the red box indicating the target

band of PALB2. Although the image is not directly presented in the manuscript, the

relevant data have been included in the statistical analysis. The specific lane distribution is

as follows: Lanes 1 and 5 are protein Markers; Lanes 2-4 represent theparacancerous

normal tissues from Patients No. 4-6; Lanes 6-8 represent the corresponding tumor group

(colorectal cancer tissues) from the above-mentioned three patients.

Normal

CRC


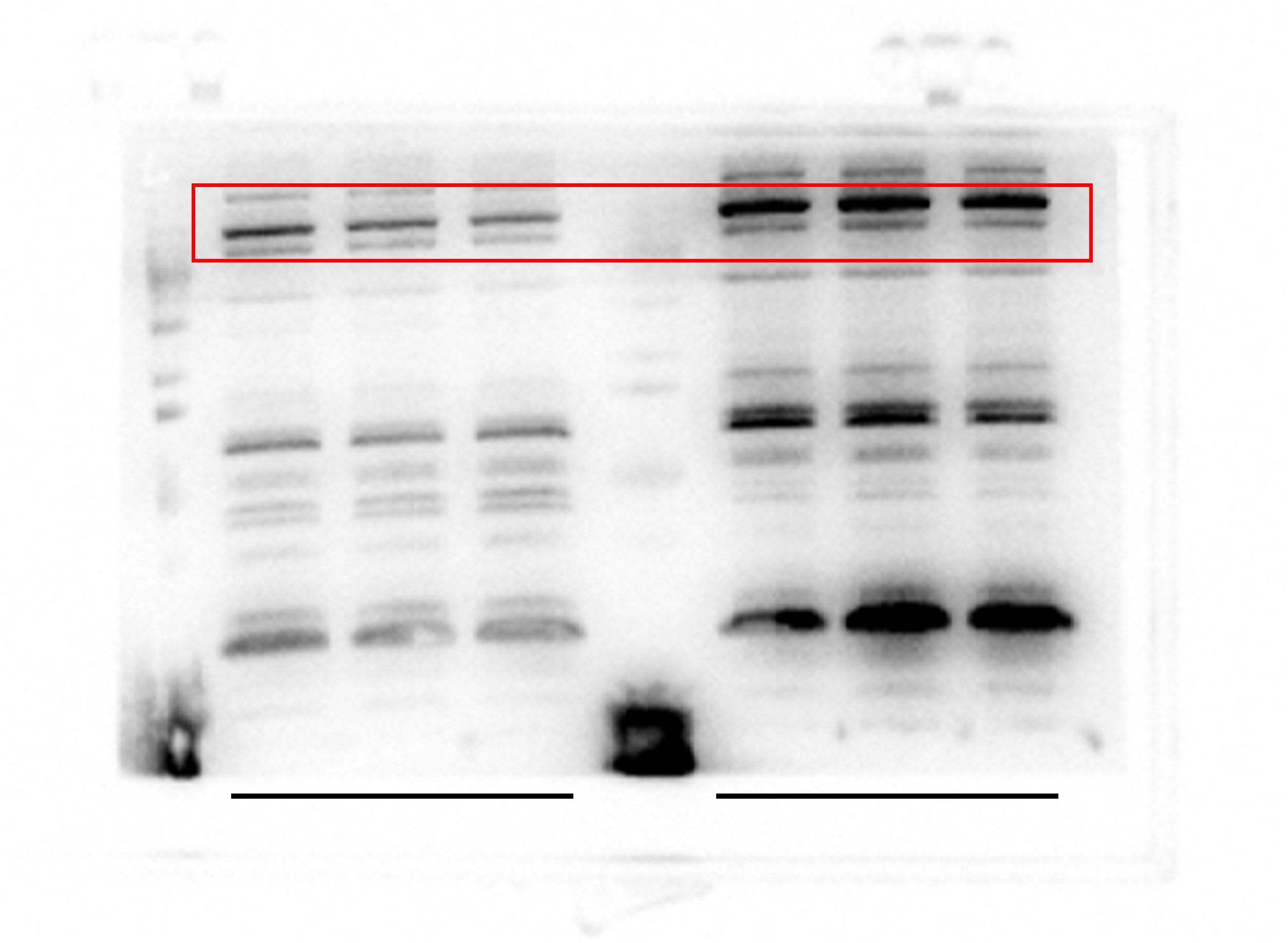


This membrane corresponds to the internal reference detection part of

the previous membrane. It was obtained by stripping the membrane

and then redeveloping it, with β-actin serving as the internal reference

protein (molecular weight: 42 kDa). The part framed in red represents

the target band of β-actin. The Marker, sample grouping, and lane

arrangement used on this membrane are all consistent with those on

the previous page.

Normal

CRC


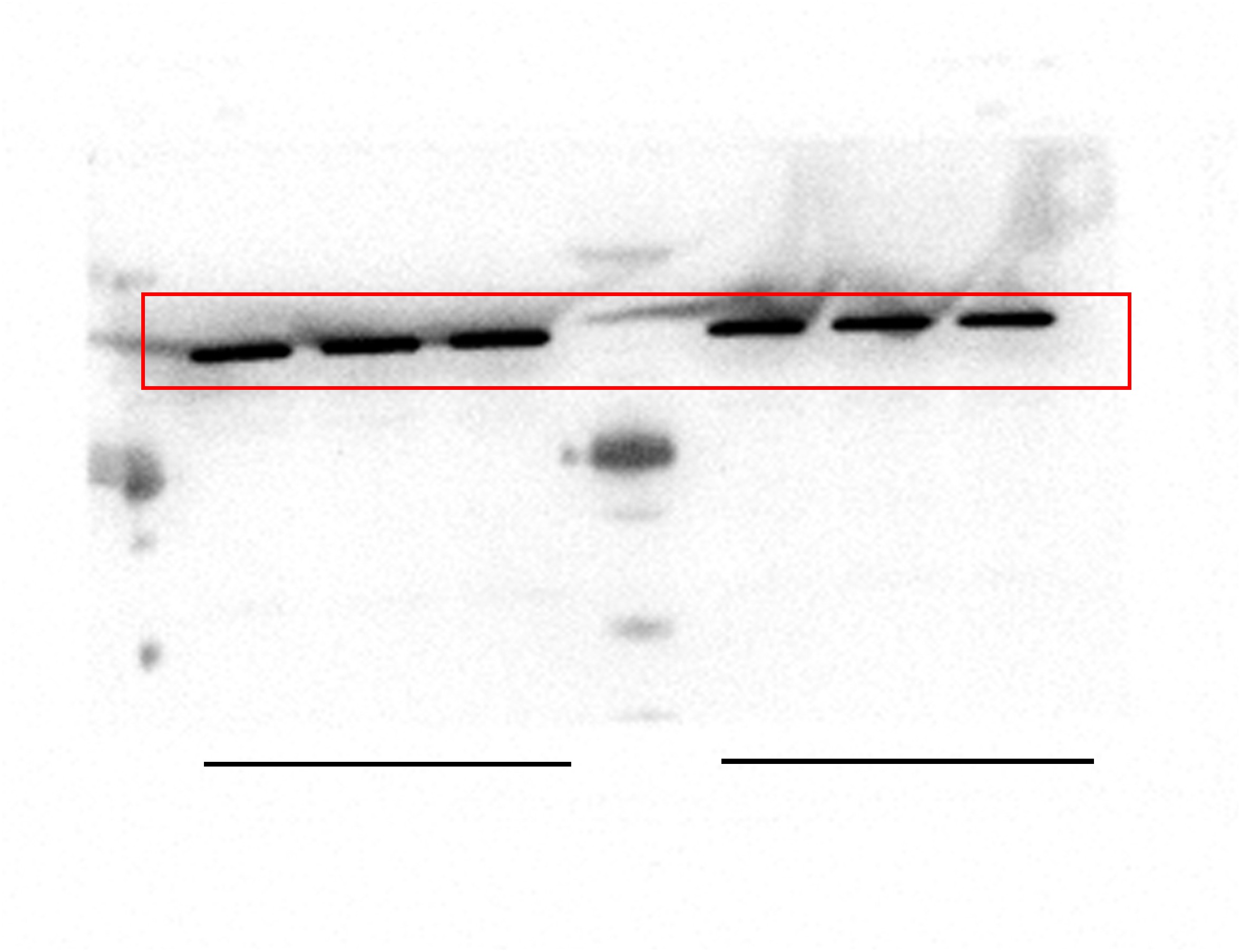


This membrane contains cancer tissues and adjacent normal tissues from 3 colorectal

cancer patients. The developed target band is HMMR with a molecular weight of 84

kDa, and the part framed in red corresponds to the third panel of Figure 8g. This image

was mirror-flipped during figure assembly. In the original image, Lanes 1, 5, 9, and 10

contain the aforementioned Marker; Lanes 2-4 belong to the colorectal cancer tumor

group (cancer tissues); and Lanes 6-7 belong to the normal control group

(paracancerous normal tissues).

Maker

Maker

Maker Maker

150 kDa

100 kDa

70 kDa

150 kDa

100 kDa

70 kDa

50 kDa

40 kDa

50 kDa

40 kDa

35 kDa

25 kDa

35 kDa

25 kDa

CRC

Normal

HMMR(84 kDa)


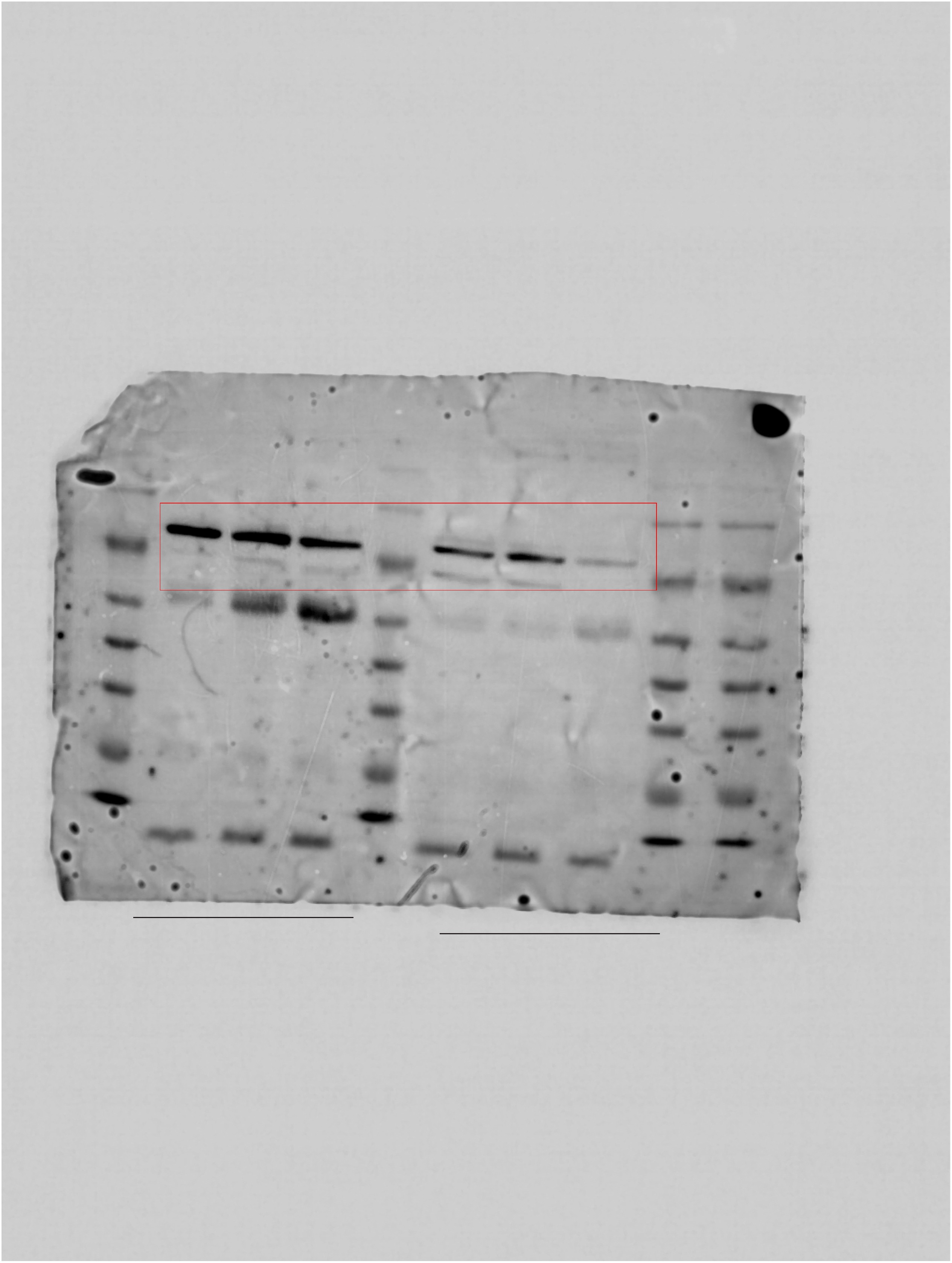


This membrane is the internal reference part of the previous

membrane, obtained by stripping and redeveloping, with β-actin

as the internal reference (molecular weight: 42 kDa)。This image

was mirror-flipped during figure assembly. The part framed in red

corresponds to the fourth panel of Figure 8g. The Marker,

groupings, and lane arrangements are consistent with those on

the previous page.

Maker

Maker

Maker Maker

150 kDa

100 kDa

150 kDa

100 kDa

70 kDa

50 kDa

70 kDa

50 kDa

40 kDa

35 kDa

40 kDa

35 kDa

25 kDa

25 kDa

CRC

Normal

β-Actin(42 kDa)


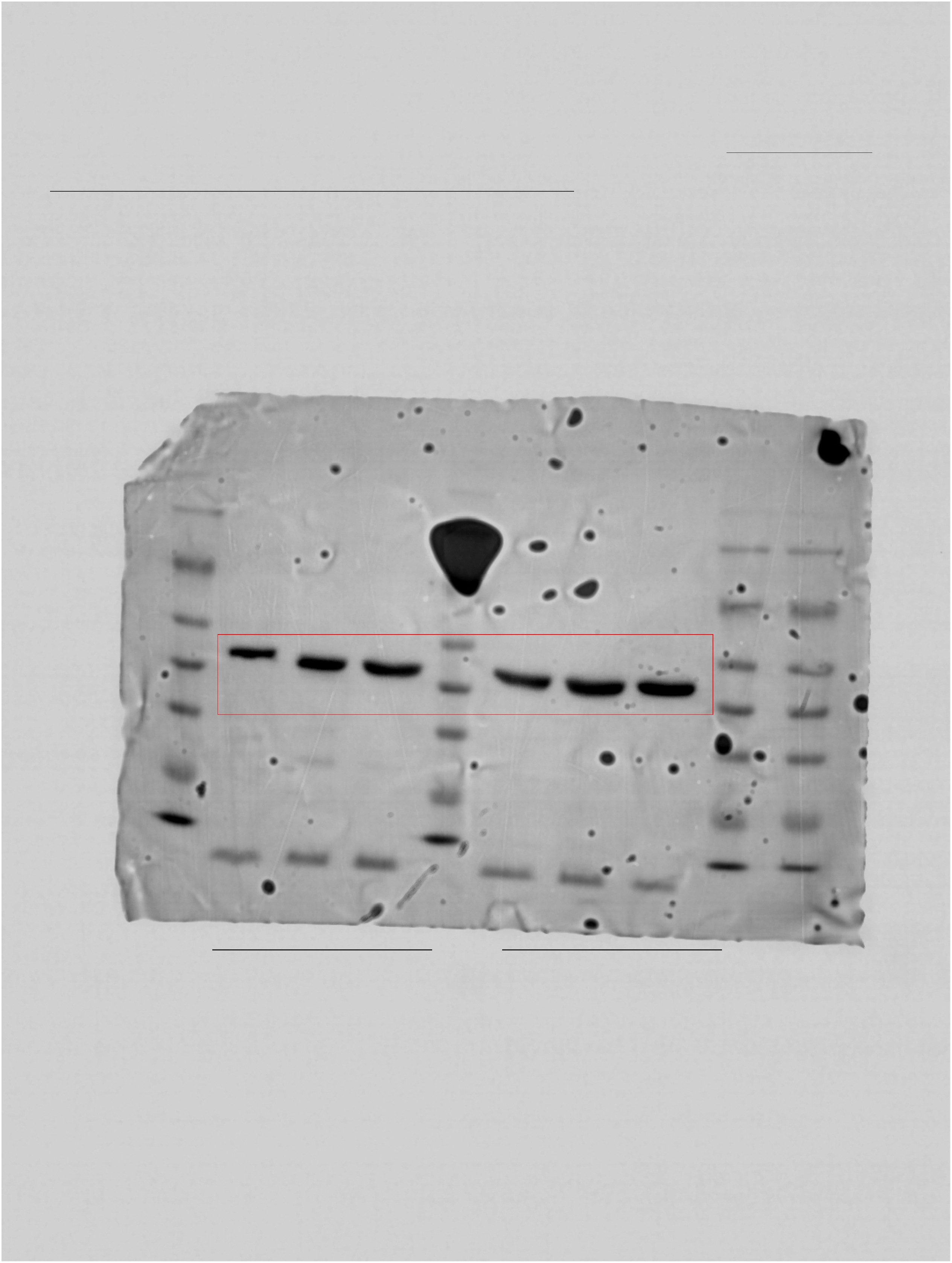


This membrane contains additional cancerous and paracancerous normal tissues from

colorectal cancer patients No. 4-6. The target band developed is HMMR (molecular

weight: 84 kDa), and the area enclosed in the red box indicates the target band of HMMR.

Although this image is not presented in the manuscript, the relevant data have been

included in the statistical analysis. The specific lane distribution is as follows: Lanes 1, 5,

and 9 are protein Markers; Lanes 2-4 are the paracancerous normal tissues from Patients

No. 4-6; Lanes 6-8 are the corresponding tumor group (colorectal cancer tissues) from the

above three patients.


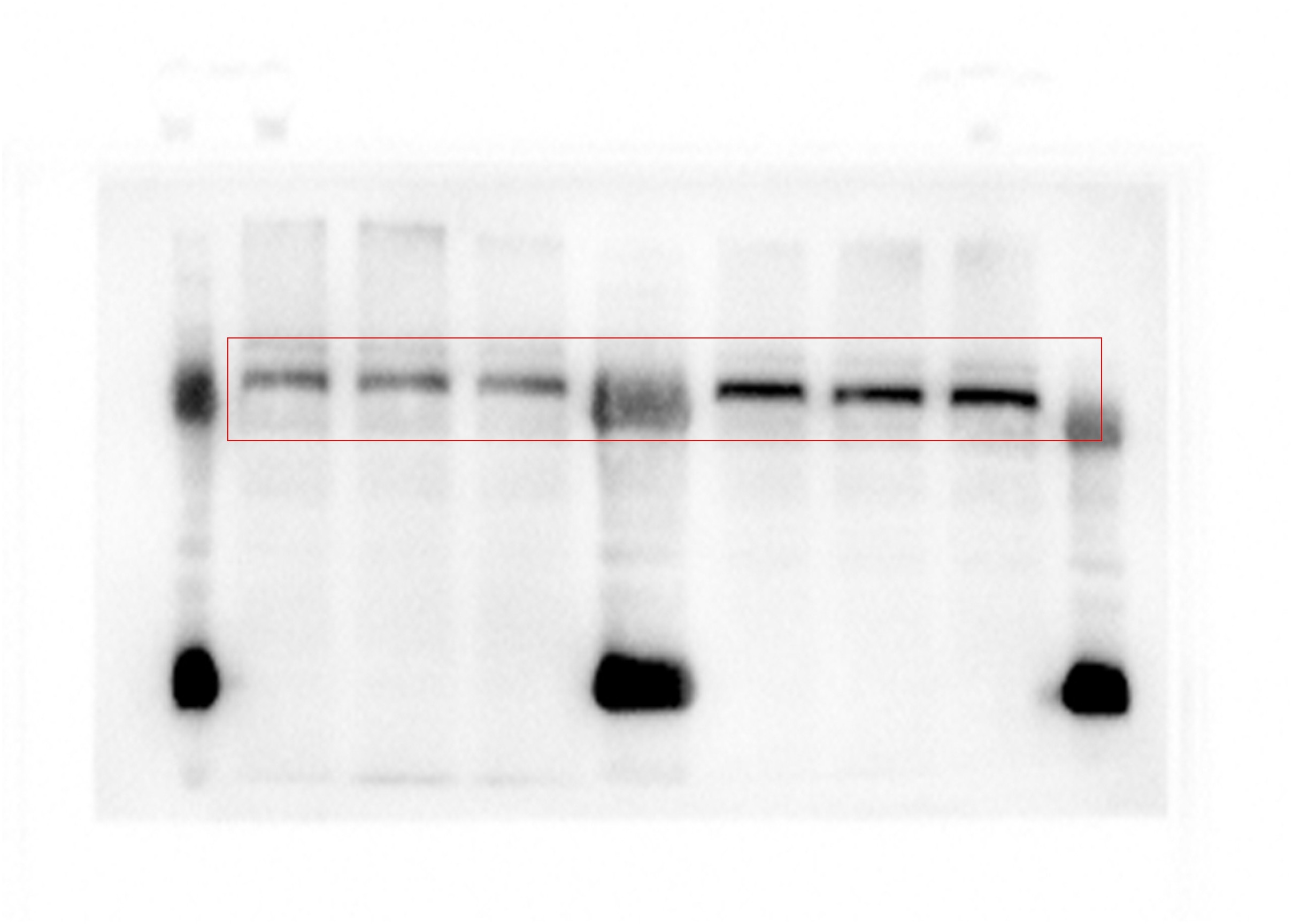


This membrane corresponds to the internal reference detection part of

the previous membrane. It was obtained by stripping the membrane

and then redeveloping it, with β-actin serving as the internal reference

protein (molecular weight: 42 kDa). The part framed in red represents

the target band of β-actin. The Marker, sample grouping, and lane

arrangement used on this membrane are all consistent with those on

the previous page.


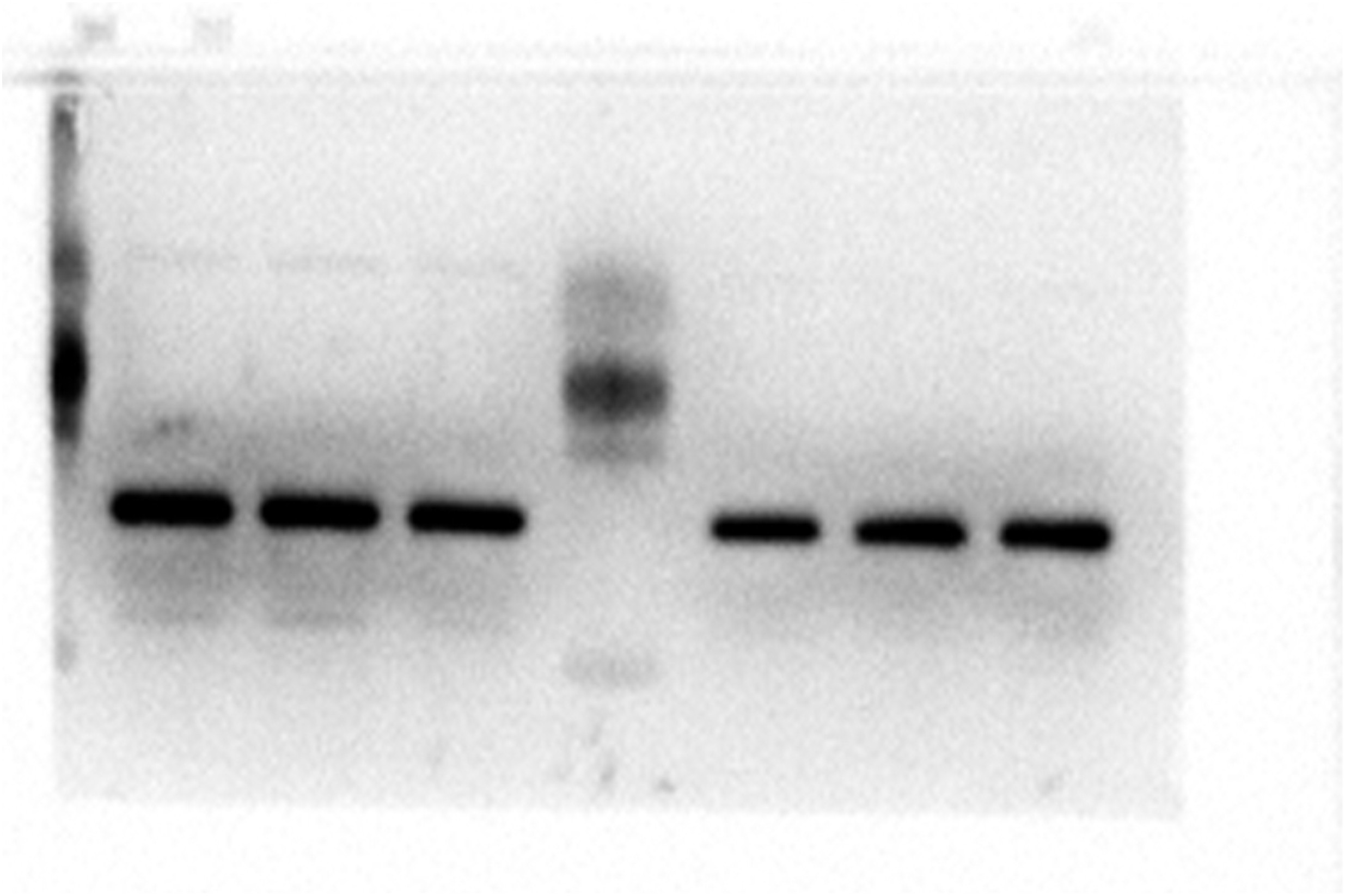


Pages 7-8 contain immunohistochemical

staining of PALB2 in adjacent normal tissues

(NC) and CRC tumor tissues(CRC),

corresponding to the top two images in Figure

9A.

Pages 9-10 show immunohistochemical

staining of HMMR in NC and CRC,

corresponding to the bottom two images in

Figure 9A.


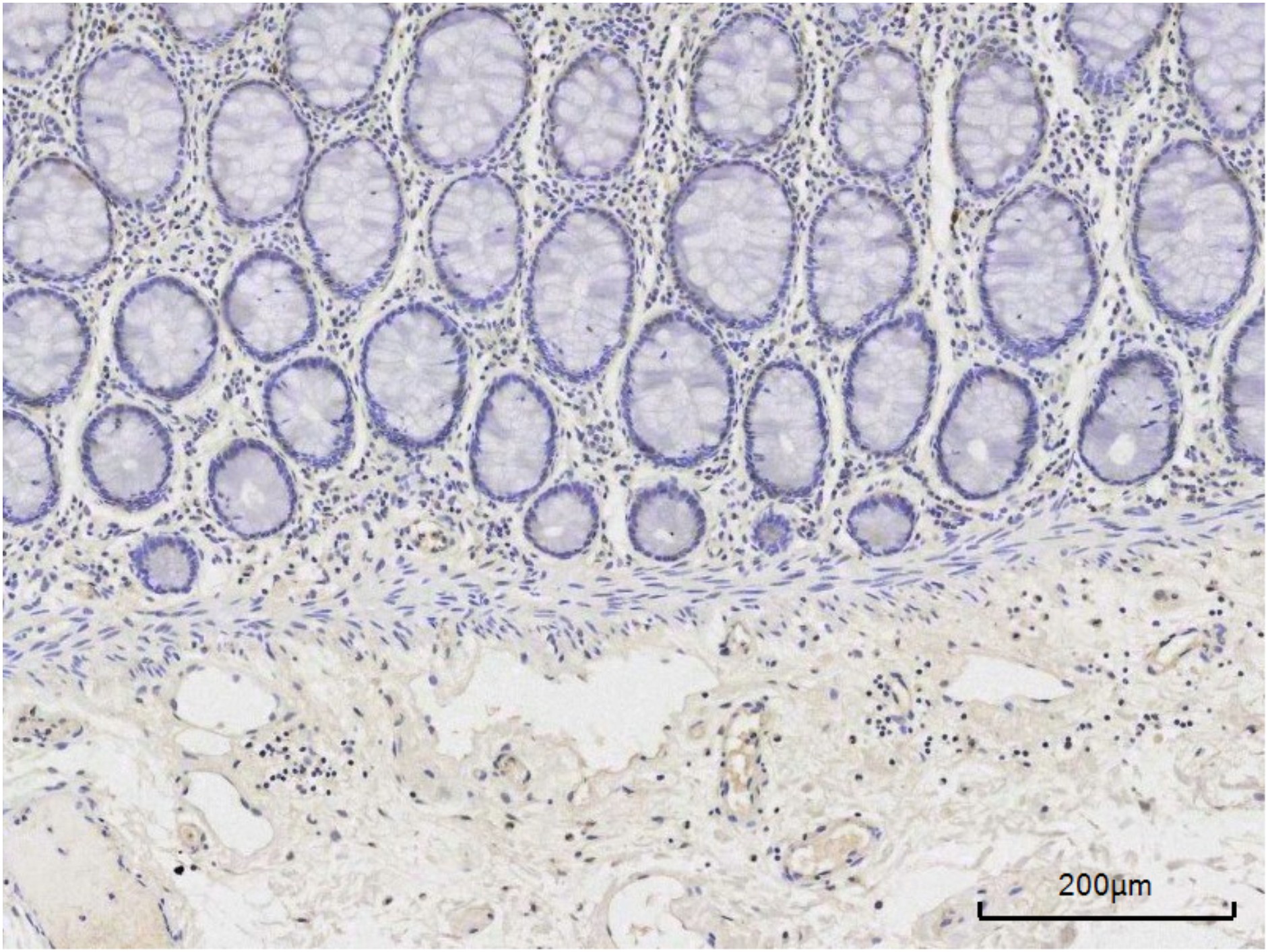

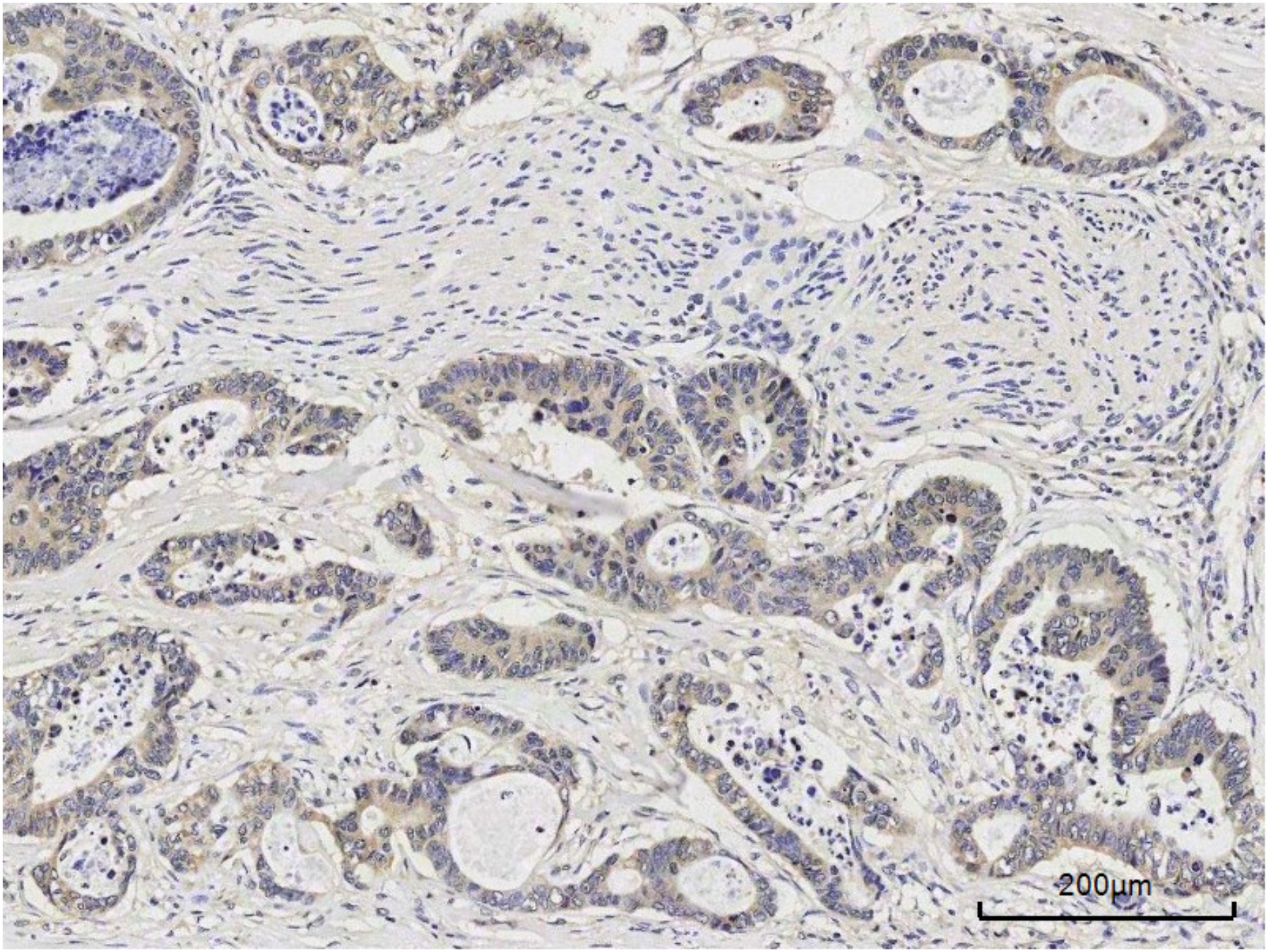

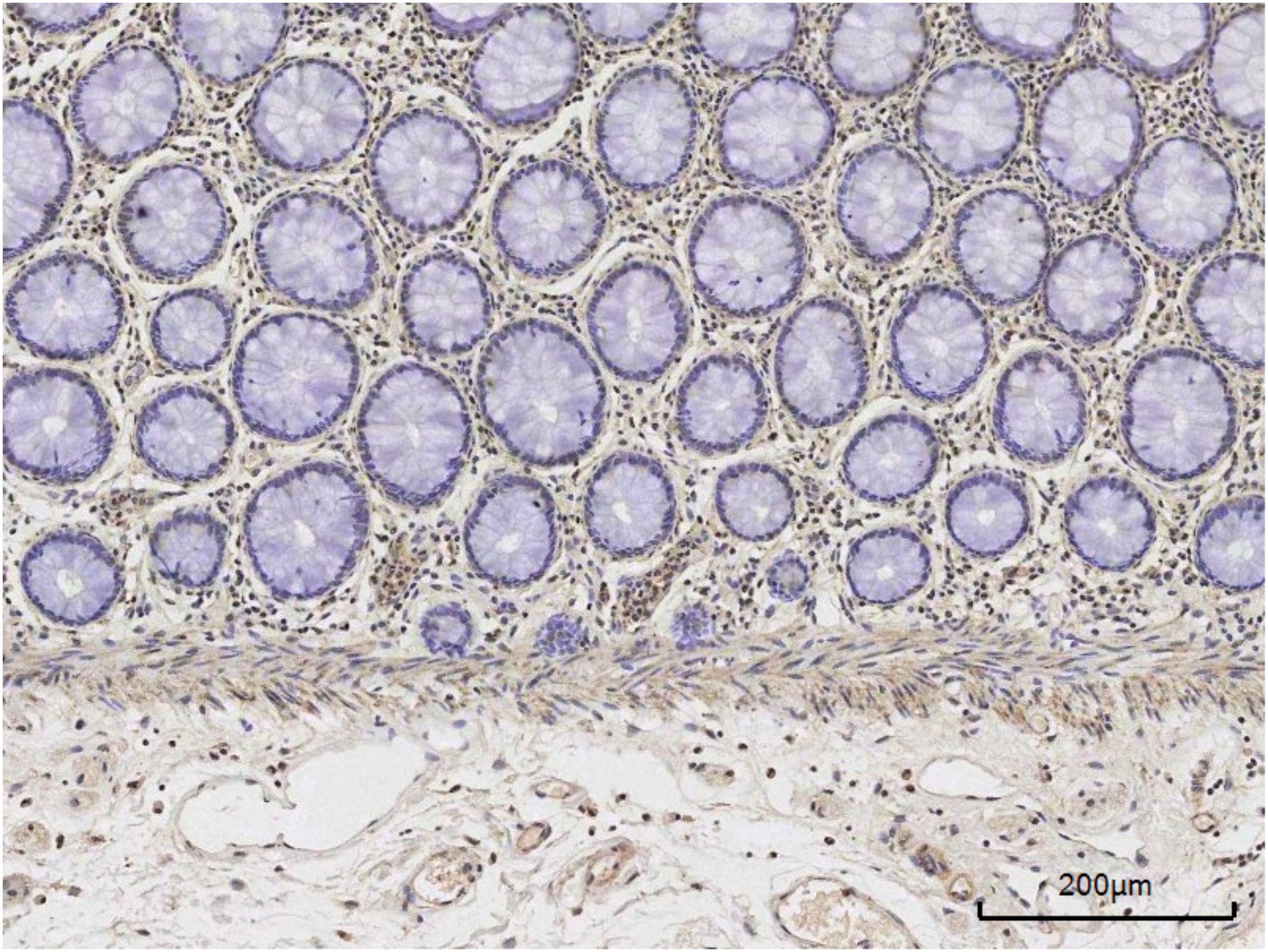

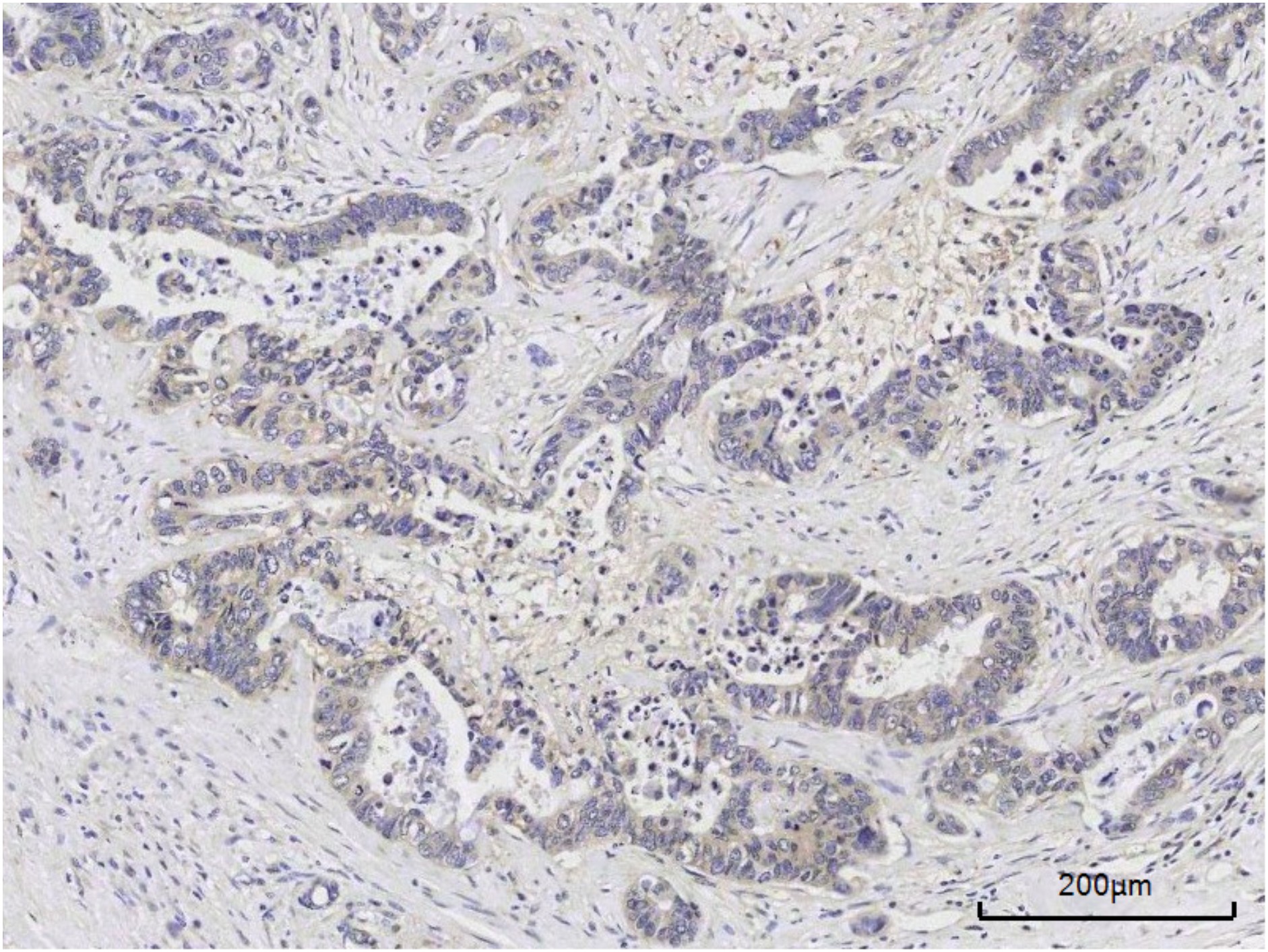


Pages 12-13 contain immunohistochemical staining of

PALB2 in normal renal vascular tissues (NC) and

atherosclerotic tissues(AS), corresponding to the top two

images in Figure 9B.

Pages 14-15 show immunohistochemical staining of

HMMR in NC and AS, corresponding to the middle two

images in Figure 9B.

Pages 16-17 present immunohistochemical staining of

PRR11 in NC and AS, corresponding to the bottom two

images in Figure 9B.

All images in this section (IHC) are the original figures

from the manuscript, without any cropping or

processing.


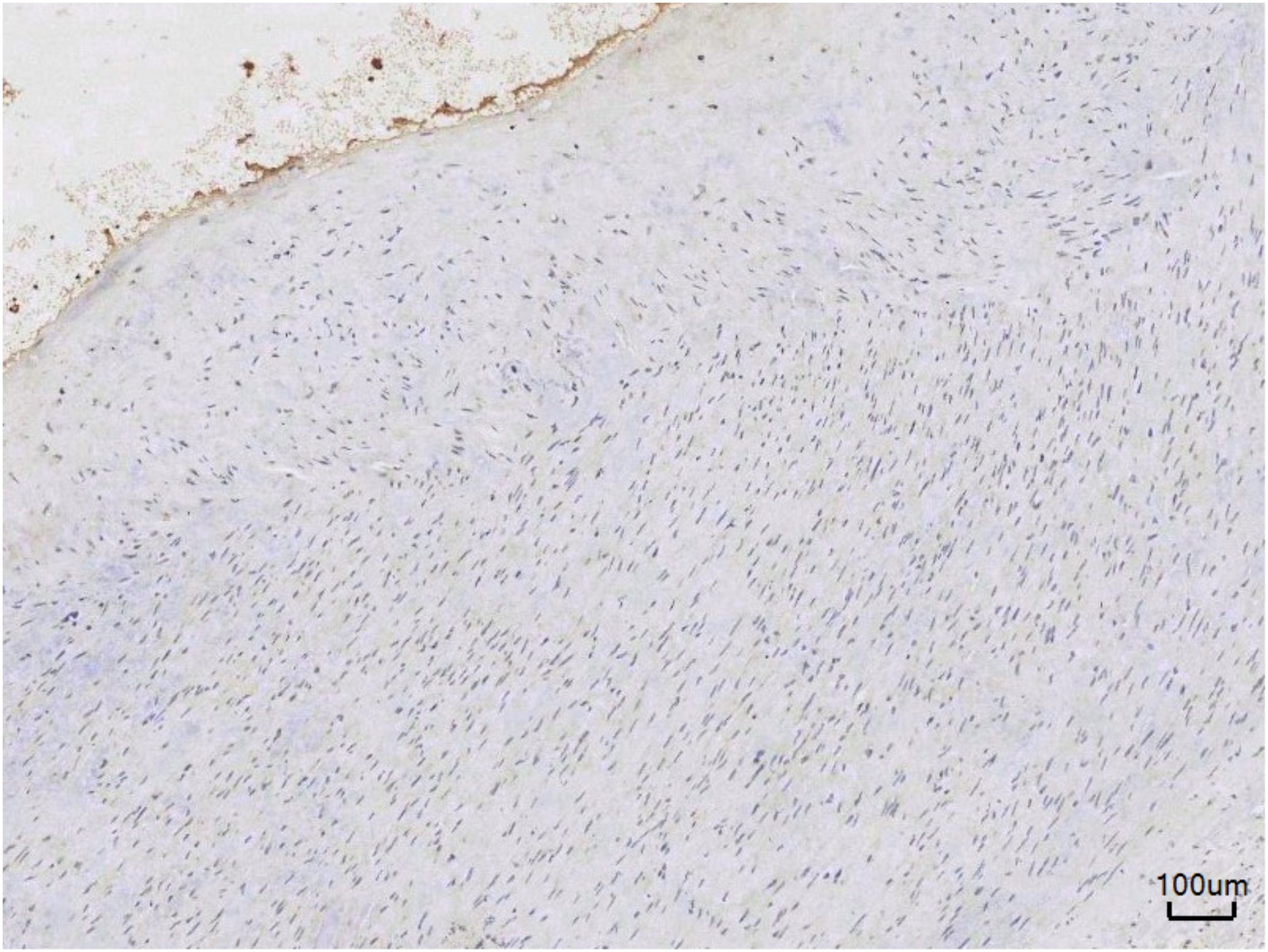

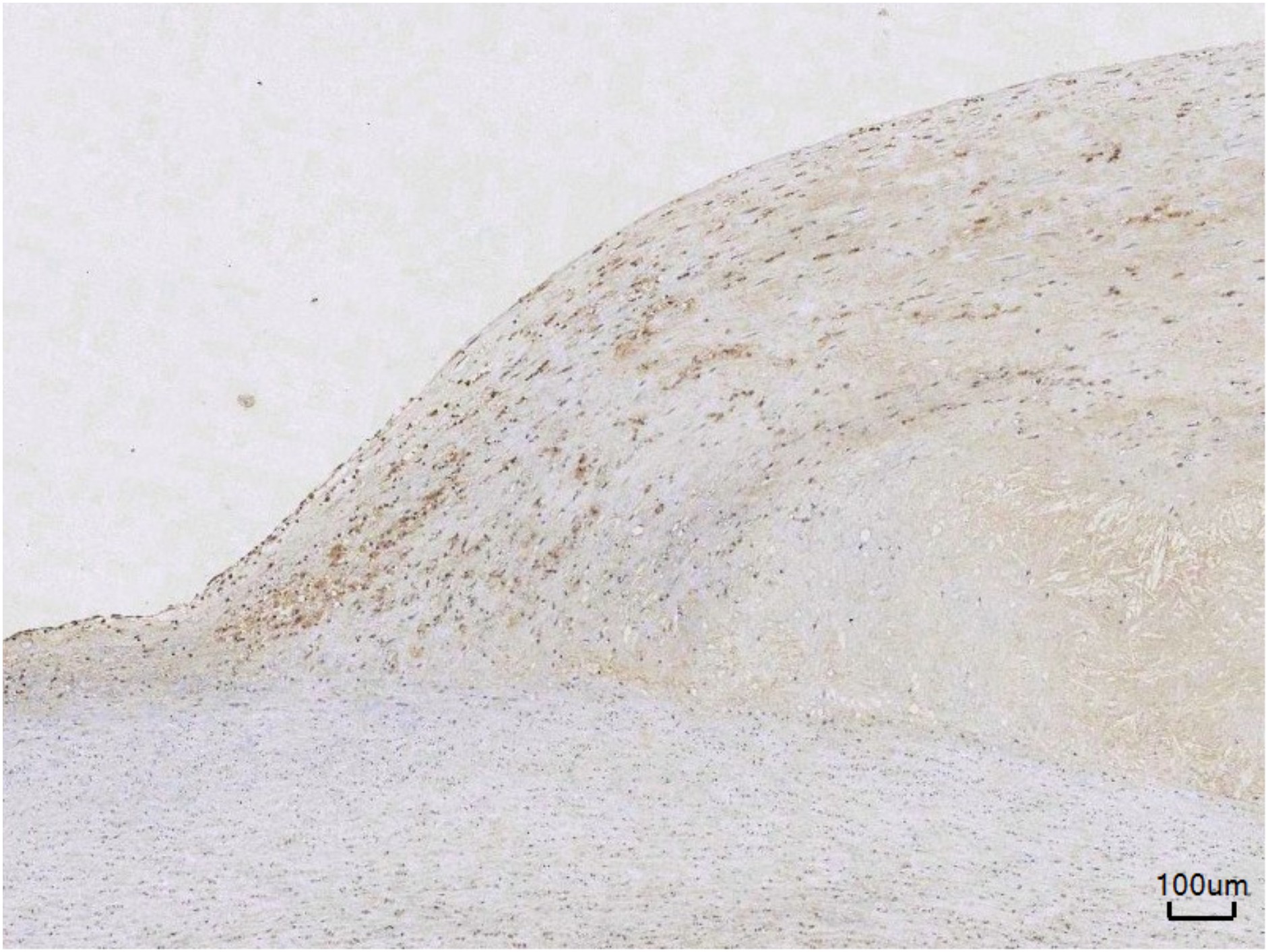

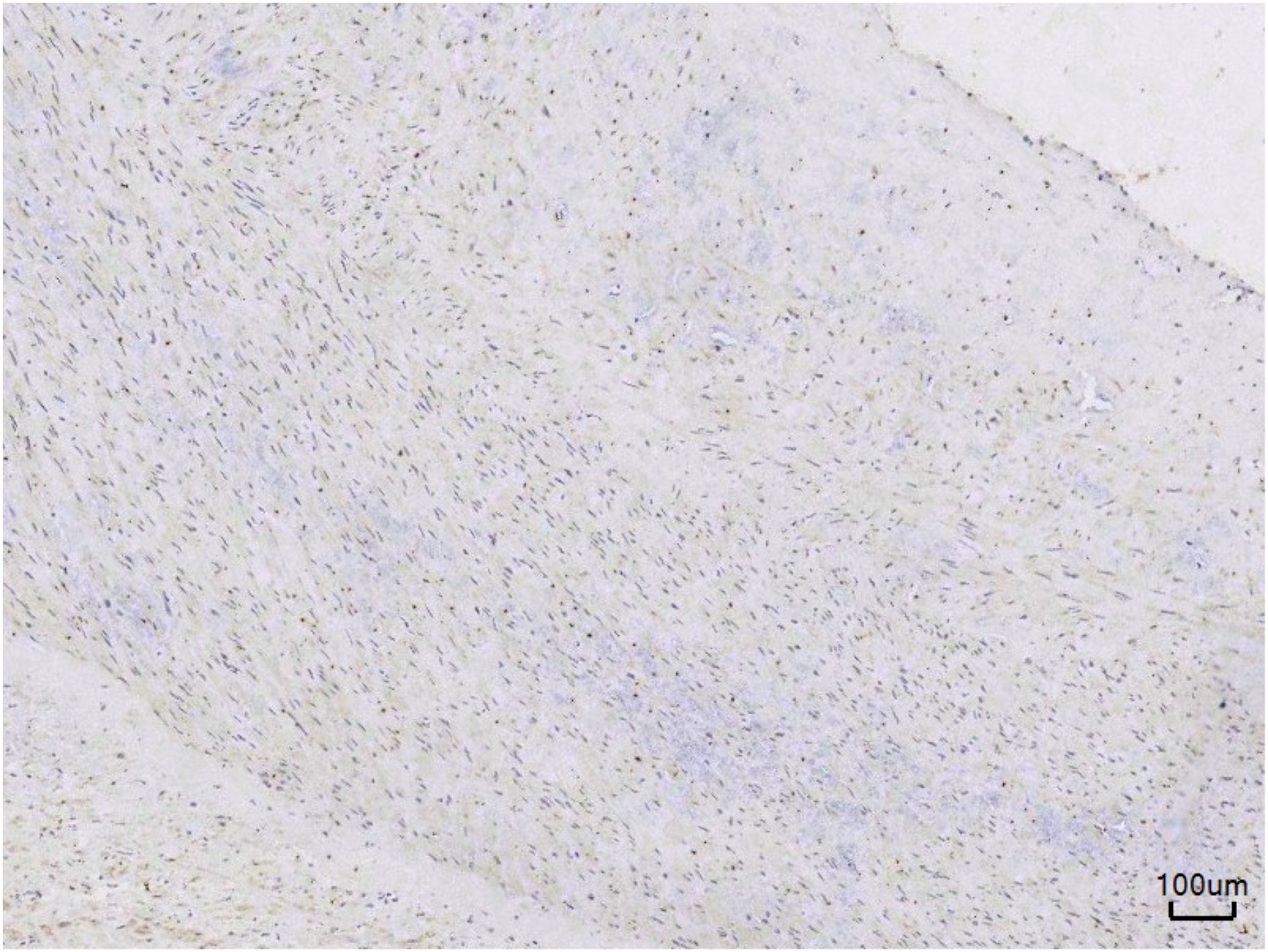

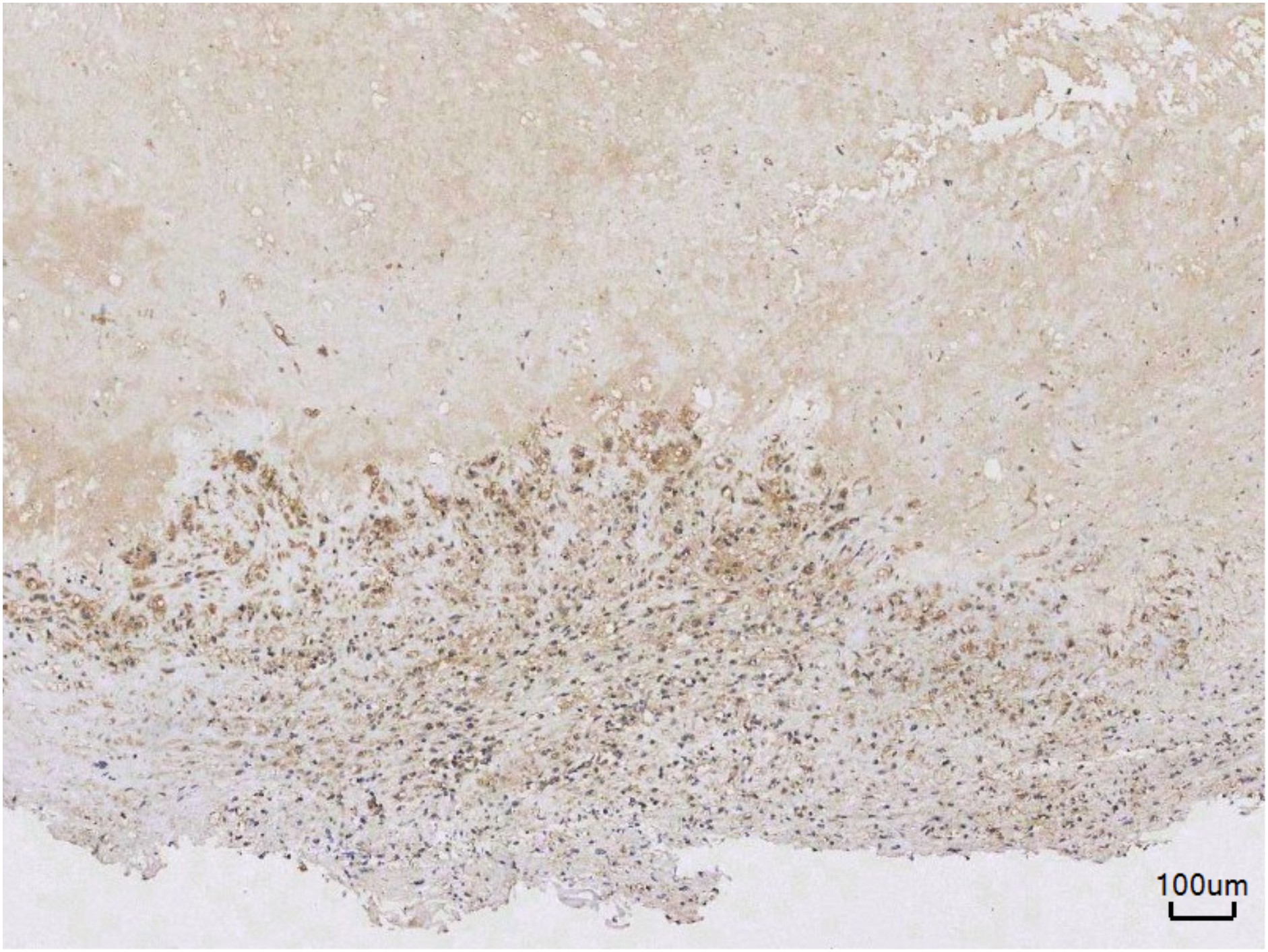

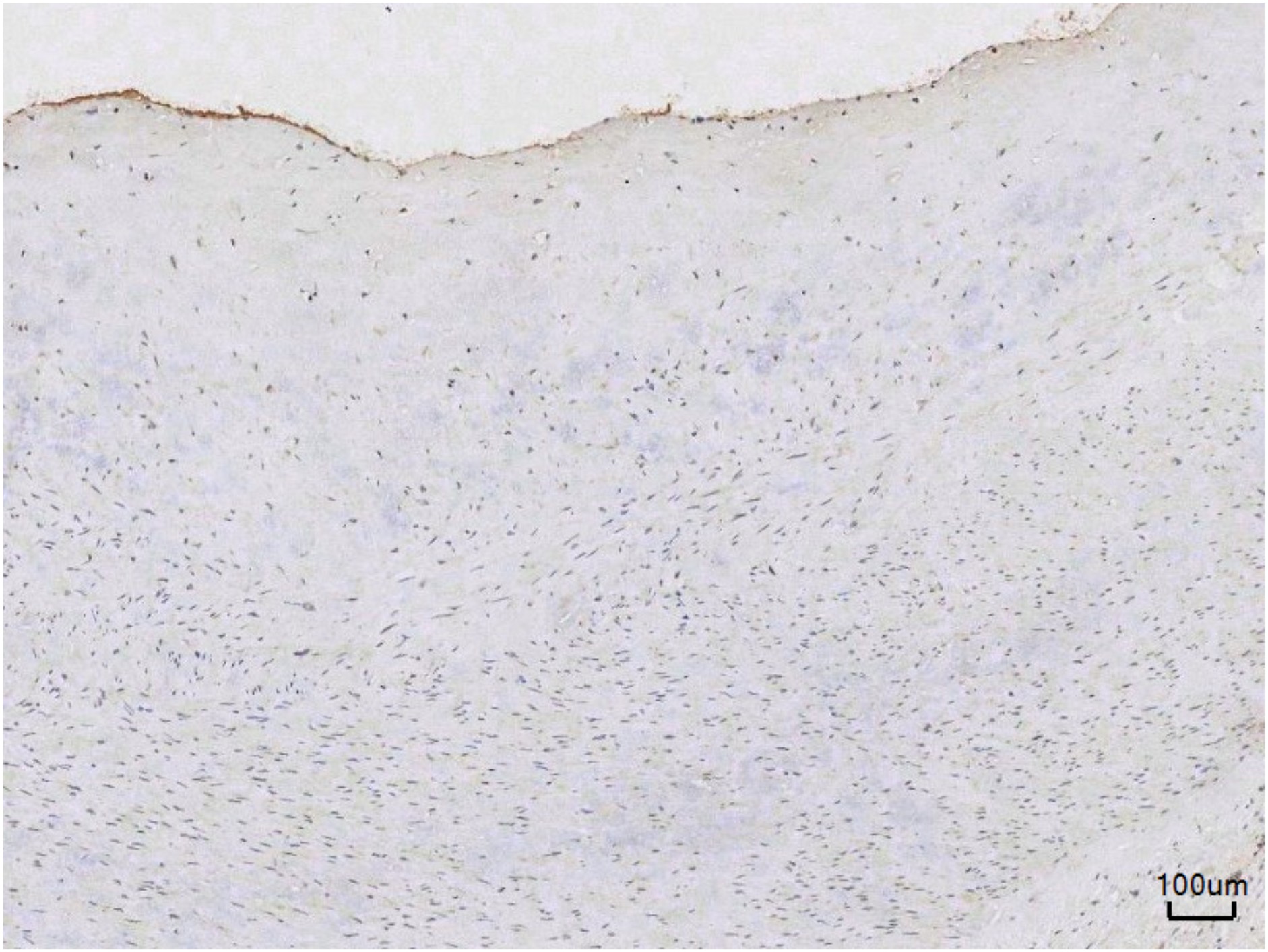

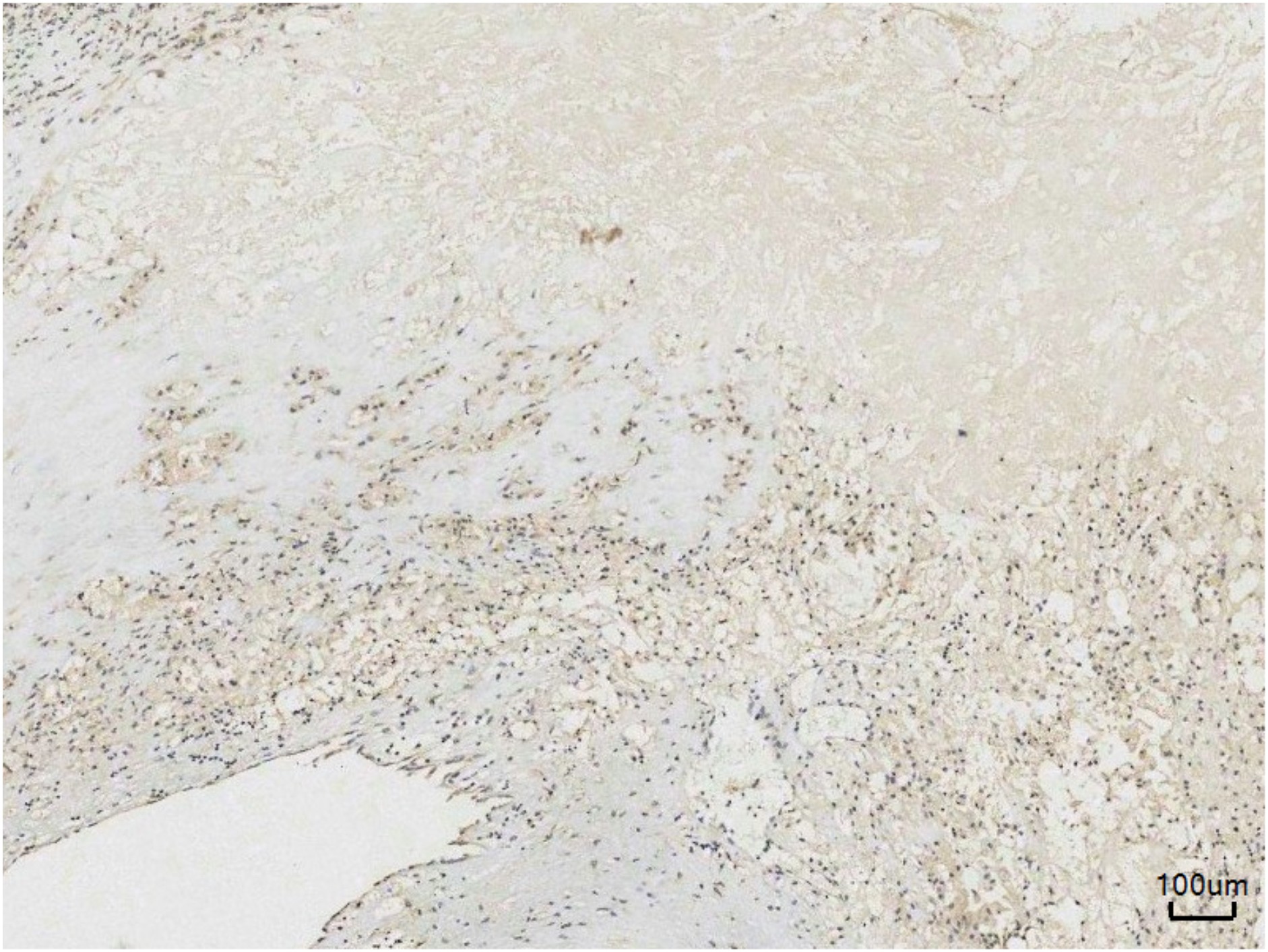

Supplement: Supplementary file 1 [file DataSheet1.docx]
