# Supplementary material for: Prognostic value and experimental validation of atherosclerosis-derived pathogenic genes in colorectal cancer
Source: Front Oncol. 2026 Jan 12;15:1728087. doi: 10.3389/fonc.2025.1728087 (PMC12832349; doi:10.3389/fonc.2025.1728087)
Supplement: Supplementary file 2 [file DataSheet2.docx]

Supplementary Tables and Figures

# Supplementary Tables

## Table S1 Summary of datasets used in this study

| Disease Type | Data Type | Source Platform | Dataset ID |
| --- | --- | --- | --- |
| Colorectal cancer (CRC) | Bulk RNA-seq transcriptome data | [TCGA (https://portal.gdc.cancer.gov)](https://portal.gdc.cancer.gov/) | TCGA-COADREAD |
|  | Expression profiling by array | [GEO (https://www.ncbi.nlm.nih.gov/gds/)](https://www.ncbi.nlm.nih.gov/gds/) | GSE87211 |
|  | Expression profiling by array |  | GSE10950, GSE39582 |
|  | Single-cell RNA-seq data |  | GSE132465 |
|  | Spatial transcriptome data |  | GSE225857 |
| Atherosclerosis (AS) | Expression profiling by array |  | GSE100927 |
|  | Expression profiling by array |  | GSE43292 |
|  | Single-cell RNA-seq data |  | GSE159677 |

## Table S2-1. Immunohistochemical Intensity, Area, and Scores of PALB2 and HMMR: Control vs. CRC Tissues

|  | *HMMR* | | | | | | *PALB2* | | | | | |
| --- | --- | --- | --- | --- | --- | --- | --- | --- | --- | --- | --- | --- |
|  | Control | | | CRC | | | Control | | | CRC | | |
|  | Intensity* | Area* | Score* | Intensity | Area | Score | Intensity | Area | Score | Intensity | Area | Score |
| Sample1 | 1 | 3 | 3 | 2 | 2 | 4 | 1 | 1 | 1 | 2 | 1 | 2 |
| Sample2 | 1 | 2 | 2 | 2 | 2 | 4 | 1 | 1 | 1 | 2 | 1 | 2 |
| Sample3 | 1 | 3 | 3 | 1 | 4 | 4 | 1 | 4 | 4 | 1 | 4 | 4 |
| Sample4 | 2 | 1 | 2 | 2 | 2 | 4 | 1 | 4 | 4 | 3 | 2 | 6 |
| Sample5 | 2 | 2 | 4 | 2 | 3 | 6 | 1 | 4 | 4 | 0 | 0 | 0 |
| Sample6 | 1 | 3 | 3 | 1 | 4 | 4 | 1 | 2 | 2 | 1 | 2 | 2 |
| Sample7 | 1 | 2 | 2 | 1 | 4 | 4 | 0 | 0 | 0 | 2 | 4 | 8 |

*Intensity: 0=negative, 1=weak, 2=moderate, 3=strong; Area: 0=0%-25%, 1=25–50%, 2=26–50%, 3=21–75%, 4=76–100%Score=Intensity×Area

.

## Table S2-2. Immunohistochemical Intensity, Area, and Scores of PRR11, PALB2 and HMMR: Control vs. AS Tissues

| *PRR11* | | | | | | | |
| --- | --- | --- | --- | --- | --- | --- | --- |
| Control | | | | AS | | | |
|  | Intensity | Area | Score |  | Intensity | Area | Score |
| Control 1 | 2 | 2 | 4 | AS 1 | 2 | 3 | 6 |
| Control 2 | 0 | 0 | 0 | AS 2 | 2 | 3 | 6 |
| Control 3 | 1 | 4 | 4 | AS 3 | 2 | 2 | 4 |
| Control 4 | 1 | 4 | 4 | AS 4 | 2 | 2 | 4 |
| Control 5 | 1 | 4 | 4 | AS 5 | 2 | 4 | 8 |
|  |  |  |  | AS 6 | 2 | 3 | 6 |
|  |  |  |  | AS 7 | 2 | 3 | 6 |

| *PALB2* | | | | | | | |
| --- | --- | --- | --- | --- | --- | --- | --- |
| Control | | | | AS | | | |
|  | Intensity | Area | Score |  | Intensity | Area | Score |
| Control 1 | 1 | 4 | 4 | AS 1 | 2 | 3 | 6 |
| Control 2 | 1 | 2 | 2 | AS 2 | 2 | 3 | 6 |
| Control 3 | 1 | 4 | 4 | AS 3 | 2 | 3 | 6 |
| Control 4 | 1 | 4 | 4 | AS 4 | 1 | 4 | 4 |
| Control 5 | 1 | 4 | 4 | AS 5 | 3 | 2 | 6 |
|  |  |  |  | AS 6 | 2 | 3 | 6 |
|  |  |  |  | AS 7 | 2 | 3 | 6 |

| *HMMR* | | | | | | | |
| --- | --- | --- | --- | --- | --- | --- | --- |
| Control | | | | AS | | | |
|  | Intensity | Area | Score |  | Intensity | Area | Score |
| Control 1 | 1 | 4 | 4 | AS 1 | 2 | 4 | 8 |
| Control 2 | 1 | 4 | 4 | AS 2 | 2 | 4 | 8 |
| Control 3 | 2 | 3 | 6 | AS 3 | 2 | 4 | 8 |
| Control 4 | 1 | 4 | 4 | AS 4 | 2 | 3 | 6 |
| Control 5 | 1 | 4 | 4 | AS 5 | 2 | 4 | 8 |
|  |  |  |  | AS 6 | 2 | 3 | 6 |
|  |  |  |  | AS 7 | 2 | 3 | 6 |

## Table S3 Primer list for hub genes in reverse transcription-quantitative polymerase chain reaction (RT-qPCR)

| Gene | Primer sequence (5'to3') |
| --- | --- |
| KPNA2-F | CTGCCCGTCTTCACAGATTCA |
| KPNA2-R | GCGGAGAAGTAGCATCATCAGG |
| CDC25C-F | ATGACAATGGAAACTTGGTGGAC |
| CDC25C-R | GGAGCGATATAGGCCACTTCTG |
| HMMR-F | AGAACCAACTCAAGCAACAGG |
| HMMR-R | AGGAGACGCCACTTGTTAATTTC |
| TKT-F | TCCACACCATGCGCTACAAG |
| TKT-R | CAAGTCGGAGCTGATCTTCCT |
| PRR11-F | GCCTCTCACTTTCAGTCCAAGC |
| PRR11-R | GTTCCAGGATGATGTTAGCCAGC |
| PALB2-F | AGGATCTCTCACCGCAGCTAA |
| PALB2-R | TCAGGCCCAACATCAAGTGTG |
| 5S rRNA-F | TACGGCCATACCACCCTGAA |
| 5S rRNA-R | TACAGCACCCGGTATTCCCA |

## Table S4 Statistical Table of Western Blot Gray Values for PALB2 and HMMR

| Normal | | | CRC | | |
| --- | --- | --- | --- | --- | --- |
| PALB2 | β-actin | PALB2/β-actin | PALB2 | β-actin | PALB2/β-actin |
| 8694.175 | 17530.569 | 0.495943686 | 9932.983 | 16271.64 | 0.610447564 |
| 5174.154 | 24004.276 | 0.215551346 | 18578.347 | 15651.861 | 1.186973677 |
| 5092.125 | 22356.894 | 0.227765315 | 21109.983 | 20999.347 | 1.005268545 |
| 12333.347 | 23228.468 | 0.530958262 | 20047.104 | 23054.347 | 0.869558526 |
| 10015.125 | 24635.468 | 0.406532768 | 22224.175 | 18709.175 | 1.187875735 |
| 867.104 | 25474.368 | 0.034038293 | 18313.347 | 14592.581 | 1.254976553 |

| Normal | | | CRC | | |
| --- | --- | --- | --- | --- | --- |
| HMMR | β-actin | HMMR/β-actin | HMMR | β-actin | HMMR/β-actin |
| 3161.25 | 22683.175 | 0.139365411 | 16956.589 | 19821.418 | 0.855468009 |
| 13326.882 | 23241.711 | 0.573403653 | 22279.054 | 17969.518 | 1.239824797 |
| 14772.539 | 16700.882 | 0.884536457 | 18426.882 | 13086.811 | 1.40804983 |
| 8434.937 | 25272.69 | 0.333756992 | 9837.381 | 13758.326 | 0.715012931 |
| 8160.397 | 24127.983 | 0.338212979 | 10620.801 | 17838.933 | 0.595371988 |
| 7301.1 | 19296.397 | 0.378365972 | 11182.833 | 15797.104 | 0.707903993 |

# Supplementary Figures
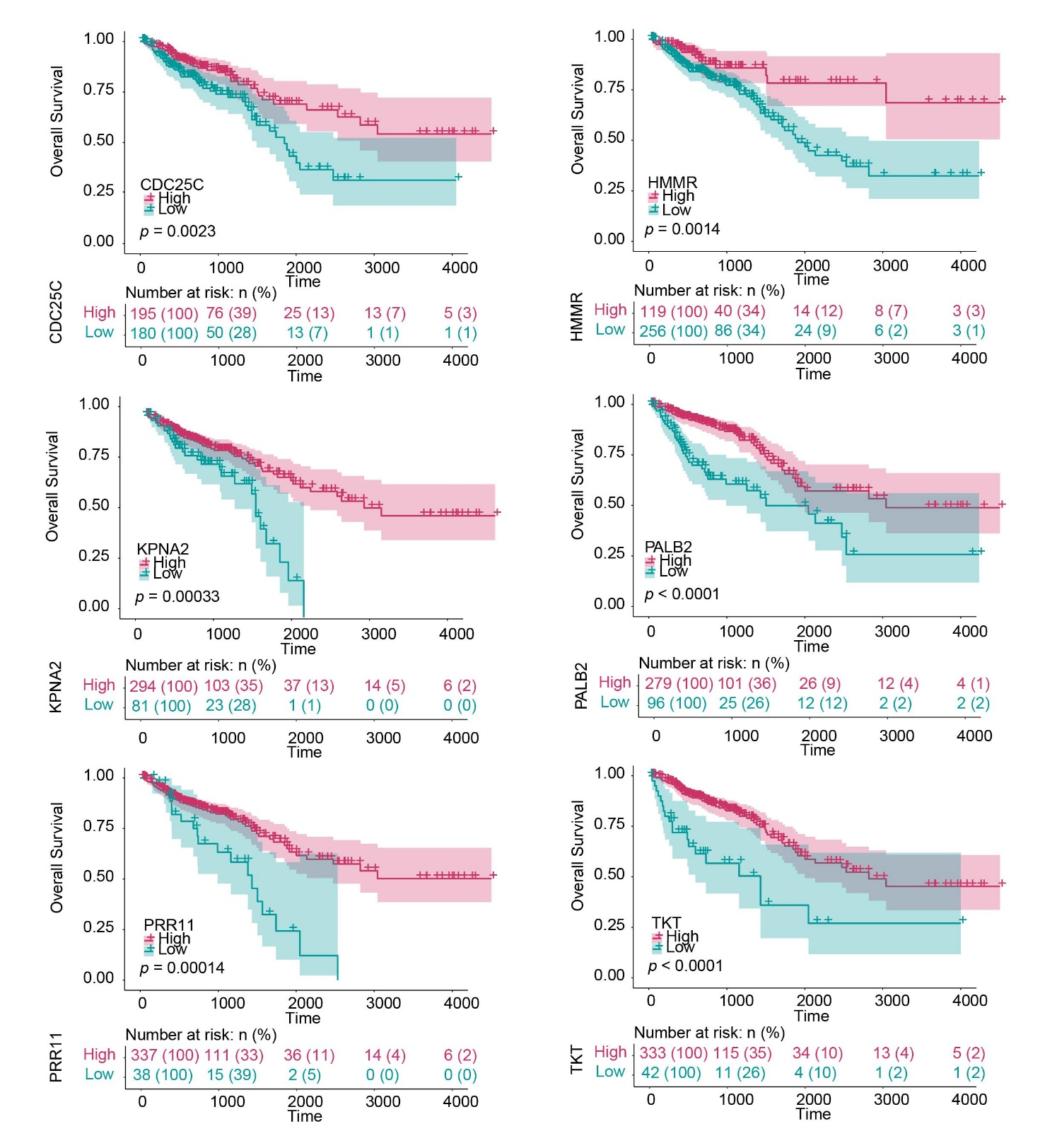


## Figure S1. Survival curve of individual modeled genes


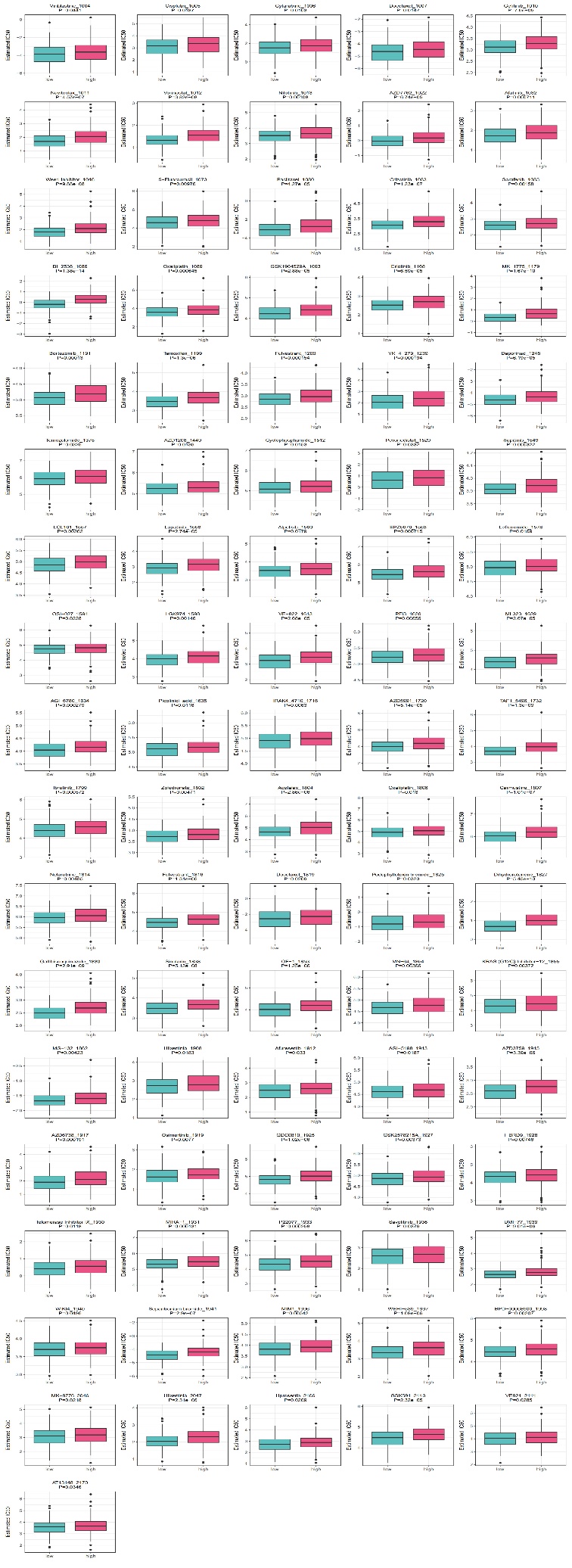


## Figure S2.  86 chemotherapeutic/Targeted therapeutic drugs with significant differences (*p*<0.05) between the high-risk group and the low-risk group.
