# Supplementary material for: Prognostic value and experimental validation of atherosclerosis-derived pathogenic genes in colorectal cancer
Source: Front Oncol. 2026 Jan 12;15:1728087. doi: 10.3389/fonc.2025.1728087 (PMC12832349; doi:10.3389/fonc.2025.1728087)

Clinical Sample Patient Information of **RT-qPCR**

**Gender Age TNM stage MLH1**

**MSH2**

**MSH6**

**PMS2**

**ERCC1**

NA

NA

NA

NA

NA

NA

+

**Ki67**

80%+

85%+

85%+

90%+

85%+

95%+

80%+

85%+

90%+

70%+

85%+

80%+

85%+

70%+

NA

80%+

90%+

80%+

70%+

80%+

85%+

80%+

80%+

Sample1 male

Sample2 male

Sample3 female 69

Sample4 female 53

Sample5 male

Sample6 female 45

Sample7 female 57

69 T3N1bM1a

+

part+

+

-

+

+

+

+

+

+

+

+

+

+

+

+

+

+

+

NA

+

+

+

+

+

+

+

+

+

+

+

+

+

+

+

+

+

NA

+

+

+

+

-

+

-

+

+

+

-

+

+

+

+

+

+

NA

+

+

+

+

+

+

+

+

51

T3N1bM0

T1N0M0

T3N0M0

T3N0M0

T3N0M0

T3N1bM0

74

+

+

Sample8 male

Sample9 male

31 T4aN1aM0

-

+

+

+

+

+

+

+

+

71

T3N0M1a

T3N0M0

T1N0M0

T3N0M0

T3N0M0

T3N0M0

Sample10 female 59

Sample11 male 60

Sample12 female 71

Sample13 male 74

Sample14 female 68

NA

NA

NA

NA

+

NA

NA

NA

NA

NA

+

Sample15 female 45 T4aN1cM1

Sample16 female 55 T4aN1aM0

NA

+

Sample17 male

Sample18 male

Sample19 female 58

Sample20 female 72

76 T3N1aM1a

+

+

+

+

58

T2N0M0

T3N1aM0

T1N0M0

+

+

+

+

+

+

+

+

Sample21 male

Sample22 male

Sample23 male

70 T4bN1bM0

+

+

+

NA

+

+

63

61

T3N0M0

T3N1aM0

+

+


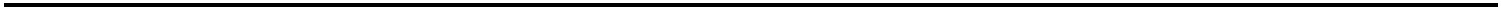

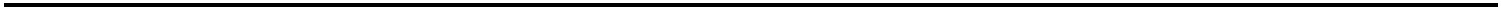

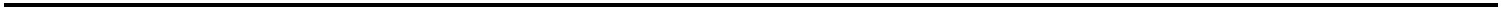


Clinical Sample Patient Information of **Immunohistochemistry Staining (IHC)**

**ID**

Sample1

**Gender Age TNM stage MLH1**

**MSH2**

**MSH6**

**PMS2 ERCC1**

**Ki67**

70%+

60%+

60%+

90%+

80%+

90%+

75%+

male

50 T4aN2bM0

+

+

+

+

+

+

+

+

+

+

+

+

+

+

+

+

+

+

+

+

+

+

+

+

+

+

+

+

+

NA

NA

NA

+

Sample2 female 76 T3N2aM1a

Sample3 female 68 T3N1aM0

Sample4 female 74 T3N2bM1a

Sample5

Sample6

Sample7

male

male

male

82

T3N0M0

63 T4aN2bM0

74 T4aN2aM1a

+

+


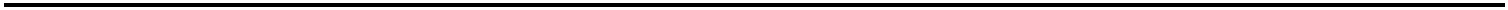

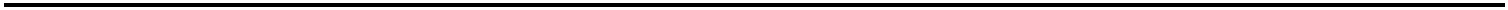

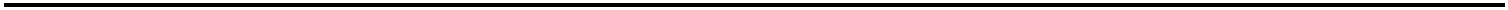


Clinical Sample Patient Information of **Western Blot (WB)**

**ID**

**Gender Age TNM stage MLH1**

**MSH2**

**MSH6**

**PMS2**

**ERCC1**

NA

+

**Ki67**

80%+

90%+

95%+

70%+

85%+

85%+

Sample1 male

Sample2 male

Sample3 female 45

Sample4 female 68

Sample5 male

Sample6 female 69

69 T3N1bM1a

+

+

+

+

+

+

+

+

+

+

+

+

+

+

+

+

+

+

+

+

+

+

+

+

71

T3N0M1a

T3N0M0

T3N0M0

NA

+

NA

NA

70 T4bN1bM0

T1N0M0


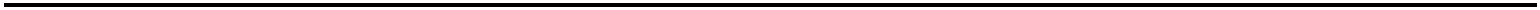

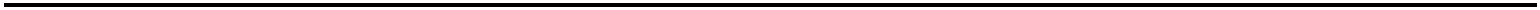

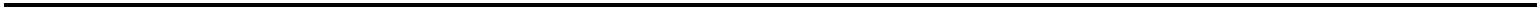

Supplement: Supplementary file 3 [file DataSheet3.docx]
